# Determinants of educational inequalities in disability-free life expectancy between ages 35 and 80 in Europe

**DOI:** 10.1016/j.ssmph.2021.100740

**Published:** 2021-01-28

**Authors:** José Rubio Valverde, Johan Mackenbach, Matthias Bopp, Henrik Brønnum-Hansen, Patrick Deboosere, Ramune Kalediene, Katalin Kovács, Mall Leinsalu, Pekka Martikainen, Enrique Regidor, Bjørn Heine Strand, Wilma Nusselder

**Affiliations:** a Department of Public Health, Erasmus Medical Center, Rotterdam, Netherlands; b Epidemiology, Biostatistics and Prevention Institute, University of Zurich, Zurich, Switzerland; c Department of Public Health, Faculty of Health Sciences, University of Copenhagen, Copenhagen, Denmark; d Department of Sociology, Vrije Universiteit Brussel, Brussels, Belgium; e Lithuanian University of Health Sciences, Kaunas, Lithuania; f Hungarian Demographic Research Institute, Budapest, Hungary; g Department of Epidemiology and Biostatistics, National Institute for Health Development, Tallinn, Estonia; h Department of Sociology, University of Helsinki, Helsinki, Finland; i Department of Public Health & Maternal and Child Health, Faculty of Medicine, Universidad Complutense de Madrid y CIBER Epidemiologia y Salud Publica, Madrid, Spain; j Norwegian Institute of Public Health, Oslo, Norway

**Keywords:** Educational inequalities, Disability-free life expectancy, Europe, Risk factors

## Abstract

Socioeconomic inequalities in disability-free life expectancy (DFLE) exist across all European countries, yet the driving determinants of these differences are not completely known. We calculated the impact on educational inequalities in DFLE of equalizing the distribution of eight risk factors for mortality and disability using register-based mortality data and survey data from 15 European countries for individuals between 35 and 80 years old. From the selected risk factors, the ones that contribute the most to the educational inequalities in DFLE are low income, high body-weight, smoking (for men), and manual occupation of the father. Potentially large reductions in inequalities can be achieved in Eastern European countries, where educational inequalities in DFLE are also the largest.

## Highlights

• Disability-free life expectancy is lower among low than the high educated in Europe.
• Over-all, the risk factor contributing most to the inequality was low income.
• Other important contributors are high body-weight, smoking and father's manual occupation.
• Size of inequality and contribution of risk factors vary importantly between countries.

## 1 Introduction

An increase in life expectancy is an important measure of population health improvement over time. However, the quality in which these years are spent is also of crucial importance (Lagiewka, 2012). Disability-free life expectancy (DFLE) is the widely used health expectancy measure, adding information on disability to life expectancy (Saito, Robine, & Crimmins, 2014). Both the EU at large and national governments aim to increase DFLE. This translates into a healthier workforce, and less exit from the labour force on the grounds of ill health, a lower burden on formal and informal care structures, leading to less strain on public finances and contributing to the longer-term sustainability of the health and social protection systems as the population ages (Lagiewka, 2012).

Educational differences in DFLE are persistent and large. Trend studies found no reduction in the socioeconomic inequalities in DFLE or even an increase in the gap (Brønnum-Hansen, Baadsgaard, Eriksen, Andersen-Ranberg, & Jeune, 2015). Educational differences in DFLE are wider than in life expectancy (Deboosere, Gadeyne, & Van Oyen, 2009; Istvan M. Majer et al., 2011a, Majer et al., 2011b). Nonetheless, we know less about the determinants of inequalities in DFLE than we know about the determinants of inequalities in life expectancy. For life expectancy, a recent study found that behavioural risk factors (obesity, physical inactivity and smoking) were important entry points to reduce educational inequalities in life expectancy in five European countries, although the magnitude of the effect of these behaviours on the inequalities varied between countries (N. E. Mäki et al., 2014). Another study including more countries and risk factors found that among the eight risk factors studied, smoking, low income and high body-weight were the most important risk factors for educational inequalities in Europe, although with large differences across countries (Anonymous, 2019).

For inequalities in disability, a study found that behavioural, work-related and living conditions account for over two thirds of the educational inequality in disability (Pérez-Hernández, Rubio-Valverde, Nusselder, & Mackenbach, 2019). Evidence on the contribution of risk factors to socioeconomic differences in DFLE is virtually absent. One study examined the contribution of fruits and vegetable consumption (Baars et al., 2019), but information on the contribution of other risk factors to socioeconomic differences in DFLE is lacking.

Furthermore, there are important differences in inequalities in DFLE across European countries and regions (Majer et al., 2011a, Majer et al., 2011b; N. Mäki et al., 2013). The development of health and social policy aimed to reduce inequalities should be tailored to the context in which it is implemented. This highlights the importance of international comparisons of determinants of inequalities in DFLE that include countries representative of the cultural, political and economic diversity of the European Union.

The aim of this study is assess the contribution of 8 risk factors to inequalities on DFLE in 15 European countries around 2010 based on a counterfactual analysis where we used for the low educated, the same prevalence of the risk factor as for the high educated. The risk factors included are father's manual occupation, low income, few social contacts, smoking, high alcohol consumption, high body-weight, low physical activity and low fruit & vegetable consumption.

## 2 Data and methods

### 2.1 Data

#### 2.1.1 Mortality

We use register-based mortality data from 15 European countries: Finland, Sweden, Norway, Denmark in the North of Europe, England & Wales, Netherlands, Belgium, Austria, Switzerland, France in the West, Spain in the South, and Hungary, Poland, Lithuania and Estonia in the East. The data covered the period between 2010 and 2014, excepting Sweden (2005–08), Norway (2006–09) and France (2004–2007). Most data covered complete national populations, excluding England & Wales, France (1% representative samples) and the Netherlands (65% population coverage). Data for most countries comes from a post-census longitudinal mortality follow-up, with the exceptions of the Netherlands (follow-up of a mix of registry data and labour force surveys) Hungary and Poland (cross-sectional unlinked studies). Appendix table A1 gives an overview of the data sources for mortality.

### 2.1.2 Disability

We used the European Union Statistics on Income and Living Conditions (EU-SILC) to obtain information on disability measured through the Global Activity Limitation Indicator (GALI). It is based on the question “For at least the past 6 months, to what extent have you been limited because of a health problem in activities people usually do?” We categorized participants with having a disability if they responded “Yes, severely” or “Yes, to some extent” to the question. We used data collected in 2010 and 2014 for most countries with the exception of Sweden, Norway and France, for which we use data for 2005 and 2009 to match the mortality data. Appendix tables B1 to B3 give an overview of the survey data used in our analyses.

### 2.1.3 Risk factors

Information on the risk factors came from the seventh round of the European Social Survey (ESS 2014), with the exception of low income which was collected from the same waves as disability from EU-SILC. This was because not all countries had information on income in ESS and EU-SILC is a more detailed source for income data.

We used two criteria to select the risk factors. The first is that reliable estimates of the relative risk of mortality and of disability were available in the literature, and the second that prevalence estimates by level of education were available from a harmonized international survey. This led to the selection of eight risk factors: father's manual occupation, low income, few social contacts, smoking, high alcohol consumption, high body-weight, low physical activity, and low fruit & vegetable consumption. This also implied the exclusion of other important risk factors like employment status and housing and work conditions because we could not find reliable relative risks in the literature. Furthermore, the need for the definition of the risk factor to be similar for the Relative Risks and the prevalence data required that exposure categories had to be dichotomized or collapsed (Appendix Table C1).

The risk factors selected comprise contrasting yet overlapping perspectives. The behavioural risk factors are located more ‘downstream’ in the causal path between education and mortality/disability than low income and father's manual occupation, which are located more ‘upstream’ than behavioural factors and partly determine why individuals with different levels of education engage in varying health-related behaviours and face other circumstance that affect health and disability, such as housing conditions and the neighbourhood where persons live (Marmot, 2003). Also, high body-weight is partly determined by diet and physical activity, and contributions of these risk factors to inequalities in mortality and disability will therefore have some overlap. In addition, father's manual occupation in part determines an individual's educational attainment (Breen & Jonsson, 2005), and in contrast to other risk factors, should not be seen as a potential mediator of education on mortality and disability, but an indicator of underlying childhood conditions on the risk of later life mortality and disability. It is important to note, however, that the validity of our results does not depend on each risk factor's position in the causal chain, and does not even depend on whether there is a causal link between low education and each risk factor. Even if there would be no such causal link, our results still inform us on what would be the impact on educational inequalities in mortality and disability of equalizing exposure to each risk factor between the low and the high educated.

Smoking was classified in three exposure categories: never (reference), former and current smokers. Income was classified in two exposure categories: lowest household income quintile versus income higher than lowest quintile (reference). Alcohol consumption was classified in three exposure categories: less than 25 g alcohol per day (reference), between 25 and 45 g alcohol per day, and more than 45 g per day. Occupation of the father was classified in two exposure categories: manual and non-manual (reference). Social contact was classified in two exposure categories: meets socially less than once a week versus meets at least once a week (reference). Fruit and vegetable was classified in two exposure categories: less than once a day fruit and vegetable consumption versus at least once a day fruit and vegetable consumption (reference). Body-weight was classified in three exposure categories: normal weight (BMI between 18.5 and <25 kg/m^2^ as reference), overweight (BMI between 25 and < 30 kg/m^2^), obesity (BMI≥30 kg/m^2^). Physical activity was classified in two exposure categories: at least 5 days a week for 30 min or longer of walking quickly, sports or other physical activity (reference) and versus less than 5 days a week minimum for 30 min or longer of these activities. Appendix table C1 gives more detail on the exposure categories and survey questions.

### 2.1.4 Education

We use the highest level of completed education as proxy for socioeconomic position: ‘low’, ‘mid’, ‘high’ corresponding to ISCED 1997 categories 0–2, 3–4 and 5–6, respectively. Our focus is on educational inequalities (not occupational or income inequalities) mainly because comparable data on educational attainment were available for both mortality and disability in all European populations under study. Since education is normally completed early in adulthood, it is also a stable measure of socioeconomic position, reducing issues with reverse causation (Daly, Duncan, McDonough, & Williams, 2002).

We restricted the analyses to ages 35–80 years because we have less reliable data on mortality by education for older ages, and for younger ages data on mortality by education were not available for all countries.

## 2.2 Methods

For descriptive purposes we calculate age-adjusted Prevalence Ratios between the high and low educated for each risk factor, by gender, and using the high educated as reference category.

We used the Sullivan method (Sullivan, 1971) to calculate partial DFLE between age 35 and 80 for each country by educational level and gender using mortality rates and disability prevalence by five-year age groups. We subtracted the DFLE of the low educated from the DFLE of the high educated to obtain inequalities in DFLE. These inequalities in DFLE are a function of 1) inequalities in mortality rates, and 2) inequalities in prevalence of disability. The inequalities in mortality and disability work in the same direction, with higher levels of either one reducing DFLE.

We used restricted cubic spline models with four knots to smooth gender and education specific prevalence of the risk factors and GALI disability prevalence across age groups, for each country.

In order to determine the contribution of risk factors to inequalities in partial DFLE we applied a method previously developed to determine the contribution of risk factors to inequalities in mortality (Nusselder & Looman, 2004). This method is based on Population Attributable Fractions (PAF) and estimates the impact of counterfactual distributions of the risk factors on the magnitude of social inequalities in health outcomes (Hoffmann et al., 2012). The PAF is defined as the fraction of deaths and disability, which would have been avoided if the prevalence of a specific risk factor had been altered to a counterfactual scenario, and is measured with Equation (1) below:

(1) $$PAF^{x}=\frac{\sum_{i=1}^{n}P_{i}\;RR_{i}^{x}-\sum_{i=1}^{n}{P_{i}}^{\prime }\;RR_{i}^{x}}{\sum_{i=1}^{n}P_{i}R{R_{i}^{x}}_{i}}$$

in which x = mortality or disability, n = number of exposure categories, $P_{i}$ = proportion of population currently in the ith exposure category, ${P_{i}}^{\prime }$ = proportion of population in the ith exposure category in the counterfactual (alternative) scenario, $\;RR_{i}^{x}$ = relative risk for the ith exposure category and for x either mortality or disability. Because we sum over all exposure categories, this gives the proportion attributed to the non-reference exposure categories (e.g. for smoking current smokers and former smokers).

We based the estimates of the contribution of risk factors on a counterfactual scenario where we set the risk factor exposure for low and medium educated to the current level of the high educated within each country (‘upward levelling’). The relative risks for mortality used in the estimation of PAFs were taken from systematic reviews, where possible taking care of selecting relative risks adjusted for confounding and based on longitudinal studies. Potential confounders were age, gender, adult socio-economic position, and other risk factors that do not lie in the causal pathway between the risk factor and mortality. The relative risks for disability were mostly based on individual studies, given systematic reviews were mostly not available. Appendix tables D1 and D2 give an overview of these Relative Risks and their information sources. For the mortality, current smokers have the highest relative risk with 2.2 relative to never-smokers. The relative risk for the obese follows with 1.7 when compared to those with normal weight. For high alcohol consumption it is 1.4 relative to those with low consumption. All other mortality relative risks are less than or equal to 1.3. In the case of disability, the relative risk for obese relative to normal weight is the highest, with a magnitude of 1.8, followed by low physical activity as well as low income with a relative risk of 1.5 and overweight with RR of 1.4. All other relative risks are less than 1.4.

We present a European average calculated as a weighted average of the values obtained for each of the 15 countries, using the total population size for each country as weight. In addition, we estimate 95% confidence intervals using bootstrapping (1000 replications).

We stratify the analyses by gender since there are important differences in mortality, disability and risk factor exposure between genders.

For sensitivity analyses, we estimated a range for our results by simultaneously increasing and decreasing the relative risks for disability and mortality for each risk factor by a fixed proportion of 20% and 40%.

All analyses were conducted using STATA version 15.

## 3 Results

### 3.1 Inequalities in disability-free life expectancy and risk factor prevalence

Table 1 shows the educational inequalities in partial DFLE between ages 35 and 80 (more extensive data included in Appendix tables E1 and E2). If no person would die or have disability, DFLE would be 45 years. We consistently observe a gradient between high and low educated, with longer DFLE for high educated than for low educated, with substantially varying levels and inequalities between high and low educated across countries.

**Table 1:** Educational Inequalities in partial disability-free life expectancy between ages 35 and 80 (in years).

		Partial disability-free life expectancy			Inequality^a^ between low level and high level [95% CI]	
		Low	Med	High		
**Men**						
*North*						
	Finland	23.5	26.4	31.7	8.1	[7.0,9.3]
	Sweden	29.7	32.5	36.9	7.2	[5.7,8.3]
	Norway	27.5	32.3	38.0	10.4	[9.1,11.7]
	Denmark	26.1	29.3	32.3	6.2	[4.8,7.9]
*West*						
	England/Wales	26.4	31.8	34.6	8.2	[7.5,9.2]
	Netherlands	26.3	29.7	33.1	6.8	[5.3,8.2]
	Belgium	24.9	31.0	34.9	10.0	[8.9,10.8]
	Austria	19.2	25.8	30.9	11.7	[10.3,13.1]
	Switzerland	25.7	30.8	34.2	8.5	[6.3,10.5]
*South*						
	France	26.4	30.2	35.0	8.6	[7.8,9.6]
	Spain	28.1	31.4	34.3	6.2	[5.2,6.4]
*East*						
	Hungary	20.1	26.4	31.5	11.5	[10.6,12.4]
	Poland	22.0	27.4	32.6	10.7	[9.8,12]
	Lithuania	19.3	26.1	32.5	13.2	[11.5,15.2]
	Estonia	16.6	22.7	27.6	11.0	[9.5,12.2]
*European mean*^b^		25.4	30.0	33.9	8.5	[8.1,8.8]
	**Women**					
*North*						
	Finland	24.4	25.8	29.3	4.9	[3.6,6.5]
	Sweden	27.1	30.8	34.0	6.9	[5.1,8.8]
	Norway	25.8	31.9	35.3	9.4	[7.8,11]
	Denmark	26.2	28.8	30.0	3.8	[2.4,5.7]
*West*						
	England/Wales	26.7	31.9	33.5	6.9	[6.1,7.9]
	Netherlands	23.8	27.3	31.3	7.4	[6.1,8.8]
	Belgium	24.3	29.9	33.6	9.3	[8.4,10.5]
	Austria	22.2	27.6	30.3	8.1	[6.9,9.6]
	Switzerland	28.2	30.9	30.7	2.6	[0.9,4.4]
	France	27.8	31.5	34.6	6.7	[5.4,7.5]
*South*						
	Spain	27.9	32.8	34.9	6.9	[6.4,7.7]
*East*						
	Hungary	20.2	28.0	32.4	12.2	[11.3,13.1]
	Poland	24.6	28.7	32.3	7.8	[6.7,8.7]
	Lithuania	21.2	27.6	34.5	13.3	[10.5,15.3]
	Estonia	18.0	23.8	29.7	11.7	[9.7,12.9]
*European mean*^b^		26.0	30.5	33.2	7.2	[6.9,7.6]

a High - low.

b Population-weighted means of all European countries in the analysis.

High educated men's DFLE ranges from 27.6 (95% CI: 26.8, 28.5) years in Estonia to 38.0 (95% CI: 37.3, 38.6) years in Norway, whereas among low educated men it varies between 16.6 (95% CI: 15.6, 17.8) years in Estonia and 29.7 (95% CI: 28.5, 30.6) years in Sweden. Inequalities in DFLE range from 6.2 (95% CI: 5.2, 7.4) years in Spain to 13.2 (95% CI: 11.4, 15.2) years in Lithuania. For the population weighted European average, DFLE for the high educated is 33.9 (95% CI: 33.6, 34.1) years, while for the low educated it is 25.4 (95% CI: 25.2, 25.7). This implies an inequality of 8.5 (95% CI: 8.1, 8.8) years.

For women, DFLE for the high educated ranges from 29.3 (95% CI: 28.6, 30.0) in Finland to 35.3 (95% CI: 34.4, 36.2) in Norway. For the low educated it ranges from 18.0 (95% CI: 16.8, 20.0) in Estonia to28.2. (95% CI: 26.9, 29.3) in Switzerland. In terms of the inequalities, it ranges from 2.6 (95% CI: 0.9, 4.4) years in Switzerland to 13.3 (95% CI: 10.5, 15.3) years in Lithuania. For the European average, the high educated DFLE is 33.2 (95% CI: 33.0, 33.5) years, while for the low educated it is 26.0 (95% CI: 25.8, 26.2) resulting in an inequality of 7.2 (95% CI: 6.9, 7.6) years.

**Fig. 1:**
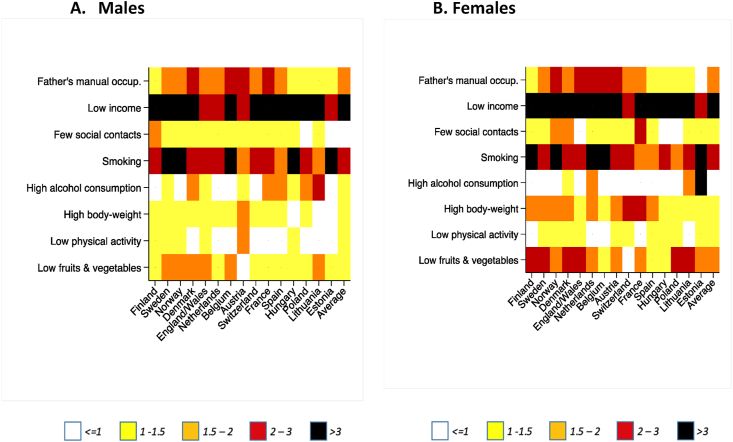
Educational inequalities in risk factor prevalence ratio for individuals between ages 35 and 80 by country and gender (prevalence rate ratio).

Educational inequalities in prevalence of risk factors are shown in Fig. 1 (detailed data in Appendix table F1). For most risk factors, the prevalence ratio is greater than 1, implying that the low educated have higher prevalence than the high educated. Exceptions include mostly physical activity and alcohol consumption in some cases, where the inequalities are often reversed. The largest inequalities are found for low income with prevalence ratios often larger than 3.0, and smoking. In the case of low income, for males the prevalence ratio is greater than 8.0 in Hungary, Poland, Lithuania and Estonia, and for females in Hungary and Poland, highlighting substantial steeper income inequality in these countries between the high and low educated.

### 3.2 Absolute effect of upward levelling

**Fig. 2:**
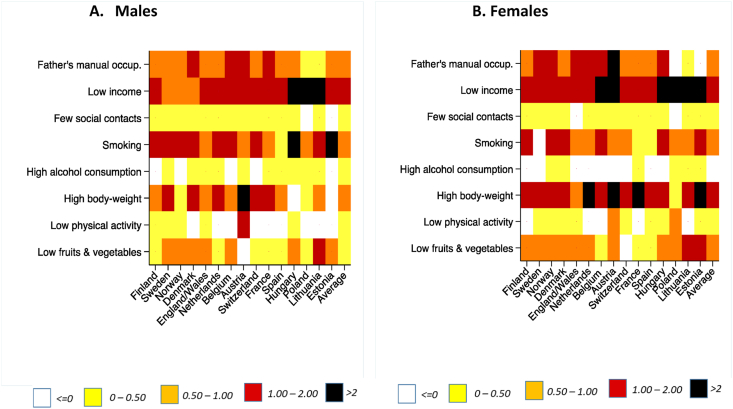
Disability-free life expectancy between ages 35 and 80 gained by low educated in ‘upward levelling’ of risk factor prevalence by country and gender (in years).

Fig. 2 and Table 2 (detailed data in Appendix table G1) present the absolute number of years of DFLE gained by the low educated through equalizing risk factors across educational groups. Equalizing the distribution of risk factors does not affect high educated, therefore disability-free years gained among the low educated also indicate the effect of ‘upward levelling’ on the inequalities in partial DFLE between low and high educated.

**Table 2:** Absolute reduction of inequality in DFLE as per ‘upward levelling’ scenario, by gender, country, risk factor (in years).

Males		Risk Factor							
Country		Father's Manual Occupation	Low income	Few social contacts	Smoking	High alcohol consumption	High bodyweight	Low physical activity	Low fruits & vegetables
*North*									
Finland	0,7		1,7	0,2	1,4	−0,1	0,9	0,4	0,4
Sweden	0,7		0,7	0,1	1,1	0,1	1,3	0,3	0,6
Norway	0,8		0,9	0,0	1,5	−0,1	0,5	0,2	0,7
Denmark	1,1		1,0	0,0	1,2	0,1	1,5	−0,4	1,0
*West*									
England/Wales	0,9		1,2	0,0	0,9	0,0	1,0	0,5	0,7
Netherlands	0,8		1,2	0,0	1,0	0,0	1,1	−0,2	0,2
Belgium	1,3		1,5	0,0	1,4	−0,1	0,6	−0,3	0,5
Austria	1,6		1,8	0,1	1,0	0,1	2,8	1,5	−0,1
Switzerland	0,8		1,7	0,1	1,1	−0,1	1,1	−0,4	0,3
France	1,2		1,2	0,1	1,0	0,1	1,1	0,0	0,3
*South*									
Spain	0,8		1,0	0,0	0,5	0,1	0,9	−0,1	0,3
*East*									
Hungary	0,7		2,4	0,0	2,4	0,0	−0,7	0,2	0,5
Poland	0,3		2,2	−0,1	0,9	0,1	0,5	−0,3	0,1
Lithuania	0,5		2,2	0,1	1,3	0,5	0,6	−0,7	1,2
Estonia	0,8		1,8	−0,1	2,3	0,0	−0,1	−0,8	0,6
Europe mean*	0,9		1,4	0,0	0,9	0,1	0,9	0,1	0,4
**Females**									

**Country**	**Father's Manual Occupation**		**Low income**	**Few social contacts**	**Smoking**	**High alcohol consumption**	**High bodyweight**	**Low physical activity**	**Low fruits & vegetables**
*North*									
Finland	0,9		1,6	0,0	1,4	0,0	1,8	−0,2	0,9
Sweden	1,2		1,1	0,0	−0,1	0,0	1,6	0,2	0,9
Norway	1,3		1,6	0,2	1,6	0,0	1,6	0,3	0,6
Denmark	0,9		1,1	0,1	1,1	0,0	1,5	0,3	0,9
*West*									
England/Wales	1,5		1,4	0,0	0,9	0,0	0,9	0,1	0,7
Netherlands	1,5		1,7	0,1	0,9	0,0	2,2	0,0	0,6
Belgium	1,6		2,1	0,1	1,3	0,0	1,1	−0,2	0,4
Austria	2,1		2,1	0,2	0,9	0,0	2,0	0,9	0,8
Switzerland	0,9		1,5	0,1	0,7	0,0	1,0	0,2	0,0
France	0,9		1,6	0,2	0,3	0,0	2,3	−0,2	0,4
*South*									
Spain	0,6		1,1	0,1	0,0	0,0	1,5	0,3	0,4
*East*									
Hungary	1,1		2,8	0,0	1,3	0,0	1,3	0,1	0,9
Poland	−0,1		2,5	0,0	0,6	0,0	0,5	0,6	0,8
Lithuania	0,2		2,2	0,1	0,8	0,0	1,7	−0,5	1,4
Estonia	−0,3		2,1	0,1	1,9	0,0	2,2	0,3	1,1
Europe mean*		1,0	1,7	0,1	0,6	0,0	1,4	0,1	0,6

Inequality is calculated as the percentage difference between the observed inequality in partial DFLE and the inequality in a counterfactual upward levelling scenario where low educated prevalence equals high educated prevalence for each risk factor. *Population weighted mean.

For men, the largest increase in DFLE for the low educated in the ‘upward levelling’ scenario occurs for low income, with increases in DFLE larger than 1 year for most countries, and greater than 2 years for some (Hungary, Poland, Lithuania, Estonia). For the European average, low income contributes 1.4 (95% CI: 1.3, 1.5) years to the observed educational inequalities in DFLE. After low income, three risk factors contribute similarly in magnitude to the educational inequalities in DFLE. These are high-body weight with 0.9 (95% CI: 0.7, 0.9) years, smoking with 0.9 (95% CI: 0.8, 1.1) years and manual occupation of the father, with 0.9 (95% CI: 0.8, 0.9) for the European average. The effect is substantially smaller for other risk factors (i.e. less than 0.5 years).

For women, the largest increase in DFLE in the ‘upward levelling’ scenario for the low educated is also found for low income, with an average contribution of 1.7 (95% CI: 1.6, 1.7) years, followed by high body-weight with 1.4 (95% CI: 1.3, 1.5) years and manual occupation of the father with 1.0 (95% CI: 0.8, 1.1) year. Smoking is not as prominent for women as it is for men, with a contribution of 0.6 (95% CI: 0.5, 0.8) years, similar to that of low fruit and vegetable consumption of 0.6 (95% CI: 0.5, 0.7) years.

There are, however, important differences between countries in the contribution of risk factors to inequalities in DFLE. Among both men and women, low income is more important in Central & Eastern Europe than in most other countries, which can, again be traced back to differences between countries in the magnitude of inequalities in low income as shown in Fig. 1 and Appendix table F1. Among men, smoking is important across all countries, but particularly so in Hungary and Estonia. For women, high body-weight contributes substantially to the inequality in France and Estonia (2 years). This also reflects lower educational inequalities in risk factor exposure for these countries, as shown in Fig. 1 and Appendix table F1.

### 3.3 Relative effect of upward levelling

Table 3 presents the contributions of the risk factors expressed as percentages of the inequality of DFLE. For the male's European average, low income accounts for 16% of the observed inequality, while three other risk factors (smoking, high body-weight and manual occupation of the father) account for around 11%. The risk factors that contribute to the inequality by 10% or more for most countries are low income, followed by high body-weight, smoking and manual occupation of the father. Countries where the impact of ‘upward levelling' on the inequality is greater than 20% are restricted to Hungary, Poland and Finland for low income, Estonia for smoking and Denmark for high body-weight.

**Table 3:** Percent reduction of inequality in DFLE as per ‘upward levelling’ scenario, by gender, country, risk factor (%).

Males	Risk Factor							
Country	Father's Manual Occupation	Low income	Few social contacts	Smoking	High alcohol consumption	High body-weight	Low physical activity	Low fruits & vegetables
*North*								
Finland	8,6	20,2	2,7	17,0	−0,9	11,3	5,2	5,3
Sweden	9,5	10,2	0,7	15,7	0,7	17,5	3,5	8,3
Norway	7,4	8,2	0,2	14,1	−0,9	4,4	1,5	6,3
Denmark	17,6	16,0	0,4	19,4	2,1	24,1	−5,9	15,7
*West*								
England/Wales	10,8	14,0	0,1	10,4	0,2	11,7	5,6	8,4
Netherlands	12,4	17,6	0,3	15,4	0,0	16,2	−2,8	3,4
Belgium	12,8	14,8	0,3	13,4	−0,9	5,5	−2,9	5,1
Austria	13,6	15,7	0,9	8,2	0,4	24,0	13,1	−0,8
Switzerland	9,6	19,8	1,6	12,5	−0,9	13,1	−4,9	4,0
France	13,4	13,9	1,1	11,2	1,3	13,2	−0,1	3,5
*South*								
Spain	12,5	16,3	0,5	7,3	1,2	15,1	−1,6	4,4
*East*								
Hungary	5,8	20,5	0,3	21,3	0,2	−6,1	2,1	4,4
Poland	3,0	20,7	−1,1	8,6	0,6	4,4	−3,1	1,2
Lithuania	3,6	16,6	0,4	10,0	3,5	4,4	−5,6	9,2
Estonia	6,9	16,5	−1,2	21,0	−0,4	−0,6	−7,0	5,3
Europe mean*	10,0	16,1	0,3	11,2	0,6	10,8	0,7	4,7
**Females**								

**Country**	**Father's Manual Occupation**	**Low income**	**Few social contacts**	**Smoking**	**High alcohol consumption**	**High body-weight**	**Low physical activity**	**Low fruits & vegetables**
*North*								
Finland	18,4	33,6	0,6	29,4	−0,1	36,3	−4,3	19,3
Sweden	16,9	15,7	0,1	−0,8	−0,3	23,7	2,2	12,8
Norway	14,0	17,2	2,1	17,0	0,0	16,9	3,6	6,3
Denmark	24,6	29,1	3,3	29,0	0,1	39,3	7,0	22,3
*West*								
England/Wales	21,1	20,5	−0,3	13,1	−0,6	13,3	1,6	10,6
Netherlands	20,3	23,3	1,1	12,5	−0,1	29,7	−0,2	7,4
Belgium	17,4	22,8	1,1	14,4	0,0	12,1	−2,0	4,1
Austria	26,1	25,4	2,1	11,6	−0,2	25,0	10,7	9,8
Switzerland	36,5	60,3	4,5	28,3	−0,8	40,0	8,1	−1,7
France	13,4	24,3	3,0	4,9	0,0	33,4	−3,1	5,3
*South*								
Spain	8,3	16,0	1,2	0,4	0,0	22,1	3,7	5,3
*East*								
Hungary	9,2	23,2	0,0	10,5	−0,2	10,7	0,9	7,6
Poland	−0,8	32,1	−0,3	8,2	0,1	6,1	7,1	10,4
Lithuania	1,8	16,3	0,5	5,8	0,3	13,1	−3,5	10,3
Estonia	−2,6	17,8	0,8	16,0	0,3	18,4	2,4	9,1
Europe mean*	13,1	23,0	0,9	8,9	−0,2	19,4	1,9	8,2

Inequality is calculated as the percentage difference between the observed inequality in partial DFLE and the inequality in a counterfactual upward levelling scenario where low educated prevalence equals high educated prevalence for each risk factor. *Population-weighted mean.

For women's European average, low income accounts for 23% of the inequality, while high body-weight around 18%, and manual occupation of the father 13% of the inequality. The impact of low income on inequalities is noticeably higher than for men, with most countries having more than a 20% reduction in the inequality in DFLE, and for Switzerland as high as 60%. It is important to note that the absolute difference in DFLE in Switzerland is the smallest of all countries with 2.5 years. High body-weight follows after low income, with for almost half the countries a reduction in inequality greater than 20%, and even around 30% for Finland and France. Again, the absolute inequalities for these two countries are among the smallest with 4.8 and 6.7 years respectively. Manual occupation of the father contributes to the inequality by more than 20% mostly in Western European countries.

### 3.4 Sensitivity analyses

**Fig. 3:**
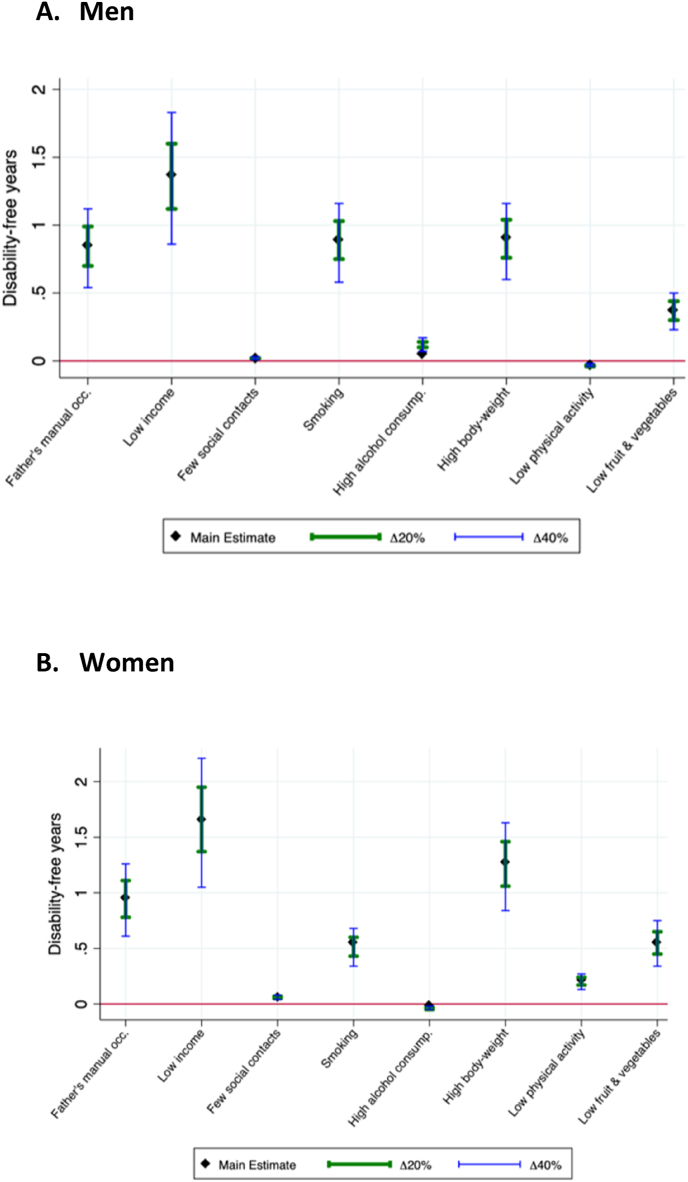
Sensitivity analyses for absolute inequality between high and low educated in Disability-free Life Expectancy (DFLE) between ages 35 and 80 for altering relative risk of mortality and disability by 20% and 40% (in years).

Fig. 3 (Appendix Table H1) shows the range of estimates for the European average obtained by increasing and decreasing the relative risks for disability and mortality by 20 and 40% for each risk factor. The importance of the factors remains unchanged within the range of estimates for the risk factors presented in Fig. 3 for both males and females.

## 4 Discussion

### 4.1 Summary of main findings

Our study examined the contribution to inequalities in DFLE of equalizing the prevalence of risk factor exposure of the low educated to the level of the highly educated. Inequalities were larger in Eastern Europe than in other region. The risk factors contributing most to inequalities in DFLE in men were first low income, followed by high body-weight, smoking and manual occupation of the father at similar levels, contributing with 1.4 and 0.9 years to the inequality in DFLE respectively for the European average. These amount to around 10–16% of the observed educational inequality in DFLE. In women most important contributors were low income, high body-weight and father's manual occupation, contributing with 1.7, 1.4 and 1.0 years for the European average. In relative terms, these amount to between 13 and 23% of the observed educational inequalities in DFLE.

The countries where the absolute impact of the risk factor upward levelling is most important are mainly Eastern European countries, particularly for low income in both genders, but also for smoking (Hungary and Estonia for men) and high body-weight (Estonia for women).

### 4.2 Interpretation and comparison with other studies

Low income was the risk factor that accounted the most for the educational inequality in DFLE. Income was characterized by strong educational inequalities, and by relative risks that are high both for disability and for mortality (1.5 and 1.3 respectively). This combination of large inequalities in exposure and excess risks of both disability and mortality explain the large effects on DFLE as both reductions in mortality and reductions in disability could increase DFLE among the low educated and thus reduce inequalities. After low income, high body-weight, manual occupation of the father, and smoking (men) accounted significantly for DFLE educational inequalities. The high contribution of body-weight to DFLE inequalities can be traced back to its higher prevalence among the low educated, combined with the highest risk for disability (1.8 for obese, 1.4 for overweight), and high relative risks for mortality (1.7 for obese). For manual occupation of the father, the contribution to the inequality is mainly explained by the differences in exposure between educational groups, since the relative risks for disability (1.3) and mortality (1.1) are not as high as for the other risk factors. Smoking in the case of men contributed substantially to the inequalities in DFLE because of its higher exposure among the low educated and its high relative risk particularly for mortality (2.2 for current smoker).

For high body-weight, low physical activity, low income and father's manual occupation the derived relative risk from the literature is higher for disability than for mortality, while for smoking and alcohol consumption, the reverse was the case. This is in line with prior studies (Reuser, Bonneux, & Willekens, 2009), where smoking is related to higher mortality and high body-weight to higher disability (and to a lesser extent to mortality), as well as with known associations of risk factor and diseases. High body-weight is associated with musculoskeletal diseases (Viester et al., 2013) and mental health afflictions (Siegel, Yancey, Aneshensel, & Schuler, 1999), contributing to disability but not as importantly to mortality. The higher relative risk of low income for disability than mortality may partly reflect the effect of financial stress on mental health (Santiago, Wadsworth, & Stump, 2011). Also we expect that there will be less inequality in access for care related to fatal conditions than to disabling conditions, in particular related to reducing the disabling consequences of diseases in terms of functioning. The lower relative risks for alcohol for disability than for mortality are expected given the known (Viester et al., 2013) strong association of alcohol with fatal hepatic disease (Becker et al., 1996), several cancers (Boffetta & Hashibe, 2006), but not with the main disabling diseases.

Physical activity is an important contributor to DFLE (Nusselder et al., 2008). The relative risk for mortality (1.3) and disability (1.5) are among the highest in our analyses. However, the prevalence of low physical activity was similar for both groups. This is reflected in a European average prevalence of 64% for the low educated and 65% for high educated males, and 68% and 67% for low and high educated females respectively. In some countries like France, Belgium and the Netherlands, inequalities in physical activity were even reversed and increased the inequalities between educational group in the upward levelling scenario. This deviant pattern as compared to the general patterns of lower physical activity among the low educated is unexpected and may be related to the threshold we used in our study.

We see differences in the importance of risk factors between countries. The reason why the contribution of a specific risk factor is larger in some countries is in part due to the magnitude of the inequality in risk factor prevalence across educational groups, as well as differences in mortality rates and disability prevalence. Countries with the largest observed educational inequality in DFLE like Lithuania, Estonia and Hungary in Eastern Europe also have large income inequalities across educational groups (with prevalence ratios of 16.0 and 10.0, with an average of 4.0 for Europe) and showed highest absolute contributions for income.

Similarly, the higher impact of smoking in countries like Estonia and Hungary can be traced back to larger inequalities in smoking prevalence (with a prevalence ratio of 3.6 and 2.8 while the European average is 2.4). For high body-weight in women, the highest contribution to inequalities occurs for women in France and Estonia; these countries show large inequalities in risk factor exposure.

To our knowledge, only one other study has examined the contribution of a risk factor, namely low fruit and vegetable consumption, to inequalities in DFLE. (Baars et al., 2019). The estimates for low fruit and vegetable consumption for 15 countries are similar to this prior study, which encompassed 10 European countries and focused solely on this risk factor. The expansion of the analysis to more risk factors and countries enables a more complete perspective on the contribution of different risk factors to the inequalities in DFLE. The contribution of low fruit and vegetable consumption has an intermediate position between factors with a small contribution (i.e. few social contacts, high alcohol consumption) and factors with a more important contribution (low income, body-weight, manual occupation of the father).

For life expectancy, a recent study using the same risk factor prevalence, mortality and relative risks for mortality found that smoking contributed the most to educational inequalities in life expectancy in Europe (19.8% for males and 18.9% females) (Mackenbach & Nusselder, 2019).

The main difference between both studies is the predominance of smoking as compared to low income and high-body weight for life expectancy, whereas for DFLE, there is a more marked contribution of low income, high body-weight and manual occupation of the father. For mortality, the relative risk for smoking is substantially larger than for the other risk factors. For DFLE the relative risks for both mortality and disability matter and for the other risk factors the relative risk for disability are larger than for mortality.

A study exploring the contribution of behavioural, work-related and living conditions to inequalities in GALI disability rather than DFLE found that these factors account for 70% of the total educational inequality in disability, with working conditions most important for men and behavioural factor more important for women (Pérez-Hernández et al., 2019). This study pointed to the importance of both behavioural and work-related factors in explaining educational inequalities in disability, using similar data of risk factor exposure from the ESS. A priori, we expected behavioural factors like high BMI, smoking and high alcohol consumption to be important also in explaining educational inequalities in DFLE. Similarly, we expected occupational factors to also play an important role in generating these inequalities, but we could not include work-related factors in our study because we could not find reliable estimates for the Relative Risk for mortality and disability for these risk factors as measured in the surveys. These factors may also contribute to the inequality in DFLE, but further research is needed to quantify this contribution.

### 4.3 Strengths and limitations

The strength of this study is that we have included a wide range of European countries and risk factors. In addition, using DFLE as summary measure of population health allows us to incorporate both mortality and disability. The validity and comparability of mortality data is also a strength of our analysis that used harmonized morality data based on individual mortality follow up, with the exception of Hungary and Poland.

Using the ESS for risk factor exposure is an advantage, given the implementation of standards in fieldwork and questionnaire design and translation. This allows for a more robust comparisons of risk factor exposure across the heterogeneous cultural contexts of European countries (Eikemo, Bambra, Huijts, & Fitzgerald, 2017). Alcohol consumption measurement in the ESS includes the use of country specific answer categories and showcards to improve measurement and that allow to estimate it in standardized units. Consumption levels were low even after we included the correction proposed by Rehm (Rehm et al., 2010) using alcohol sales. It is likely that we have underestimated alcohol consumption and that focusing on alternative drinking patterns like binge drinking, could show larger inequalities between educational levels.

Measuring disability and income using EU-SILC has some advantages, including the large number of Europeans surveyed. The data from the Nordic countries and the Netherlands comes from administrative registers for several variables, that are supplemented with household interviews (Iacovou, Kaminska, & Levy, 2012), improving the quality of the income data. Furthermore, there is variation in how countries ask the GALI question, ranging from slightly changing the phrasing of the standard GALI question to asking the question in parts (Berger et al., 2015). Finally, EU-SILC excludes the institutionalized population. In general the effect of missing the institutionalization on DFLE is limited as either the prevalence is small, or the excess disability in the institutionalized population is low, and most institutionalization occurs after the age of 80 (Cambois, Jagger, Nusselder, Van Oyen, & Robine, 2016, pp. 207–229).

Our approach based on population attributable fractions assumes the effects of the risk factors are causal, but the relative risks for mortality and disability that were obtained from the literature, were based on observational studies which cannot determine causality. In addition, in particular for disability as reviews or meta-analyses were virtually absent, and individual studies were sometimes small and measures of disability varied between the studies and was not based on the GALI indicator. This constitutes a limitation, considering it would be ideal to use relative risks from literature based on our disability instrument. However, prior research has shown strong associations between the GALI indicator and other disability measures, like Activities of Daily Living (ADLs) and mobility (Berger et al., 2015; Cabrero-García, Juliá-Sanchis, & Richart-Martínez, 2020; Van Oyen, Bogaert, Yokota, & Berger, 2018), so we used these in the absence of better estimates.

Using the same relative risk across different countries, educational groups, age and gender is a limitation of our study. Our measurement of low income captures a more qualitative rather than quantitative dimension, considering that the threshold for belonging to the lowest income quintile varies substantially by country (from around 2.800 in Estonia to 21.900 in Denmark). There are also important differences in social protection schemes between countries that might safeguard against the effect of factors like low income on mortality and disability in different ways. This in contrast with smoking, that is expected to have a more consistent effect on mortality across diverse populations. Furthermore, there might be differences in relative risks across educational groups, for instance in smoking (Schaap et al., 2008) and alcohol consumption (Mäkelä & Paljärvi, 2008), that might impact more adversely the low educated.

There is heterogeneity in the response rates reported in the ESS, ranging from 44% in England and Wales to close to 70% in the case of Lithuania. High non-response may in particular bias the results when the low response is product of a systematic loss of respondents (Lance & Vandenberg, 2009), but we lack information whether this is to be expected.

Because the risk exposure data used is cross-sectional, we were unable to analyse duration of exposure and to account for delays that could feasibly occur between exposure to a given risk factor and a fatal or disabling outcome. This implies that an under (or over) estimation of the risk factor impact on inequality in DFLE can be the result of using recent prevalence data, in particular when there is a long delay between exposure and the health outcome, as is the case for smoking and mortality. For instance, in countries which were more advanced in the smoking epidemic, like England & Wales, inequalities in smoking were smaller in the past (Giskes et al., 2005), we may have overestimated the current contribution of smoking on inequalities in DFLE and our results could more precisely reflect the contribution of smoking in the future (Kulik et al., 2013).

Furthermore, it would be desirable to analyse risk factors in combination, however, available methods to do so assume that they are mutually independent. This cannot be assured in our study, since ‘downstream’ risk factors (i.e. high-body weight, smoking, physical activity) are determined in part by ‘upstream’ factors like income, as well as by other risk factors. This is the case for body-weight being determined in part by diet, as well as physical activity. This implies that the contribution of all risk factors together may be less than the sum of the individual effects, but the total effect is indeed much larger than each of the separate effects.

We have assumed that the direction of causality runs from risk factor to DFLE. However, the causal chain could be reversed, for instance physical inactivity could be the cause of obesity, but obesity can also cause physical inactivity. Furthermore, we have used education as our variable to estimate the contribution to educational inequalities in DFLE, but it is feasible that education also impacts some of the risk factors we have selected, particularly in the case of income, as well as engagement in health related behaviours.

There is the possibility that there are overlapping contributions of risk factors to the educational inequalities in DFLE. For this reason the contribution in our study cannot be added to obtain the cumulative effect.

Although we included a large number of countries, because of the lack of mortality data available for several large countries like Germany, and countries with large inequalities like Romania and Bulgaria, we are unable to include them in the study. The inclusion of these countries would improve the European average estimates presented in this study.

### 4.4 Policy implications

Our results highlight the importance of addressing income inequality between socio-economic groups. This is substantial in all European countries, but is rather marked in Eastern Europe. Policy options include progressive taxation and social security provisions which are fundamental in sheltering vulnerable individuals from the negative impact of poverty and low income.

Secondly, our results point to the importance of addressing inequalities in the prevalence of high-body weight. Policy options include incentivizing changes in dietary patterns (e.g., through subsidies to healthy foods and taxes to unhealthy ones), as well as incentivizing participation in physical activity, especially for low socio-economic position individuals.

Thirdly, our results indicate that early-life interventions that support healthy growth and development for children in lower socioeconomic position families (e.g., through free pre-school programs) could aid in reducing inequalities in DFLE later on in life.

Finally, equity-focused tobacco control policies, which include measures that have a larger impact among lower socioeconomic position smokers (e.g., raising the price of cigarettes) remain fundamental tools that policymakers have at their disposal to curb the demand for this unhealthy behaviour in the population.

## 4.5 Conclusions

The risk factors that contribute the most to the educational inequalities in DFLE are low income, high body-weight, smoking (for men) and manual occupation of the father. Potentially large reductions in inequalities can be achieved in Eastern European countries, where the educational inequalities in DFLE are also the largest.

## Funding

This study was conducted as part of the LIFEPATH project, which has received financial support from the European Commission (Horizon 2020 grant number 633666), and as part of the project “Longer life, longer in good health, working longer? Implications of educational differences for the pension system”, which has received financial support from Netspar (Network for Studies on Pensions, Aging and Retirement). Data were partly collected as part of the DEMETRIQ project, which also received support from the European Commission (grant number FP7-CP-FP grant no. 278511). The funders had no role in study design, data collection and analysis, decision to publish, or preparation of the manuscript.

Pekka Martikainen was funded by the Academy of Finland and MINDMAP, a European Commission HORIZON 2020 research and innovation action grant 667661.

## Declaration of competing interest

The authors declare to have no competing interests.

## Author statement

José Rubio Valverde: Data curation, Methodology, Formal analysis, Writing – original draft. Johan Mackenbach: Conceptualization, Writing – original draft, Supervision, Project administration, Funding acquisition. Matthias Bopp: Data curation, Writing – review & editing. Henrik Brønnum-Hansen: Data curation, Writing – review & editing. Patrick Deboosere: Data curation, Writing – review & editing. Ramune Kalediene: Data curation, Writing – review & editing. Katalin Kovács: Data curation, Writing – review & editing. Mall Leinsalu: Data curation, Writing – review & editing. Pekka Martikainen: Data curation, Writing – review & editing. Enrique Regidor: Data curation, Writing – review & editing. Bjørn Heine Strand: Data curation, Writing – review & editing. Wilma Nusselder: Conceptualization, Methodology, Writing – original draft, Supervision, Project administration, Funding acquisition

## References

[bib1] Baars A.E., Rubio-Valverde J.R., Hu Y., Bopp M., Brønnum-Hansen H., Kalediene R., White C. (2019). Fruit and vegetable consumption and its contribution to inequalities in life expectancy and disability-free life expectancy in ten European countries. International Journal of Public Health.

[bib2] Becker U., Deis A., Sorensen T.I., Gronbaek M., Borch‐Johnsen K., Muller C.F., Jensen G. (1996). Prediction of risk of liver disease by alcohol intake, sex, and age: A prospective population study. Hepatology.

[bib3] Berger N., Van Oyen H., Cambois E., Fouweather T., Jagger C., Nusselder W., Robine J.-M. (2015). Assessing the validity of the global activity limitation indicator in fourteen European countries. BMC Medical Research Methodology.

[bib4] Boffetta P., Hashibe M. (2006). Alcohol and cancer. The Lancet Oncology.

[bib5] Breen R., Jonsson J.O. (2005). Inequality of opportunity in comparative perspective: Recent research on educational attainment and social mobility. Annual Review of Sociology.

[bib6] Brønnum-Hansen H., Baadsgaard M., Eriksen M.L., Andersen-Ranberg K., Jeune B. (2015). Educational inequalities in health expectancy during the financial crisis in Denmark. International Journal of Public Health.

[bib7] Cabrero-García J., Juliá-Sanchis R., Richart-Martínez M. (2020). Association of the global activity limitation indicator with specific measures of disability in adults aged below 65. The European Journal of Public Health.

[bib8] Cambois E., Jagger C., Nusselder W., Van Oyen H., Robine J.-M. (2016). International comparisons of disability prevalence estimates: Impact of accounting or not accounting for the institutionalized population *international Measurement of disability*.

[bib9] Daly M.C., Duncan G.J., McDonough P., Williams D.R. (2002). Optimal indicators of socioeconomic status for health research. American Journal of Public Health.

[bib10] Deboosere P., Gadeyne S., Van Oyen H. (2009). The 1991–2004 evolution in life expectancy by educational level in Belgium based on linked census and population register data. European Journal of Population/Revue européenne de Démographie.

[bib11] Eikemo T.A., Bambra C., Huijts T., Fitzgerald R. (2017). The first pan-European sociological health inequalities survey of the general population: The European social survey rotating module on the social determinants of health. European Sociological Review.

[bib12] Giskes K., Kunst A.E., Benach J., Borrell C., Costa G., Dahl E., Mackenbach J.P. (2005). Trends in smoking behaviour between 1985 and 2000 in nine European countries by education. Journal of Epidemiology & Community Health.

[bib13] Hoffmann R., Eikemo T.A., Kulhánová I., Dahl E., Deboosere P., Dzúrová D., Mackenbach J.P. (2012). The potential impact of a social redistribution of specific risk factors on socioeconomic inequalities in mortality: Illustration of a method based on population attributable fractions. Journal of Epidemiology & Community Health.

[bib14] Iacovou M., Kaminska O., Levy H. (2012). Using EU-SILC data for cross-national analysis: Strengths, problems and recommendations.

[bib15] Kulik M.C., Menvielle G., Eikemo T.A., Bopp M., Jasilionis D., Kulhánová I., Mackenbach J.P. (2013). Educational inequalities in three smoking-related causes of death in 18 European populations. Nicotine & Tobacco Research.

[bib16] Lagiewka K. (2012). European innovation partnership on active and healthy ageing: Triggers of setting the headline target of 2 additional healthy life years at birth at EU average by 2020. Archives of Public Health.

[bib17] Lance C.E., Vandenberg R.J. (2009). Statistical and methodological myths and urban legends: Doctrine, verity and fable in the organizational and social sciences.

[bib18] Mackenbach J.P., Valverde J.R., Nusselder W. (2019). Determinants of inequalities in life expectancy in Europe. Lancet Public Health.

[bib19] Majer I.M., Nusselder W.J., Mackenbach J.P., Kunst A.E. (2011). Socioeconomic inequalities in life and health expectancies around official retirement age in 10 Western-European countries. Journal of Epidemiology & Community Health.

[bib20] Majer I.M., Nusselder W.J., Mackenbach J.P., Kunst A.E. (2011). Socioeconomic inequalities in life and health expectancies around official retirement age in 10 Western-European countries. Journal of Epidemiology & Community Health.

[bib21] Mäkelä P., Paljärvi T. (2008). Do consequences of a given pattern of drinking vary by socioeconomic status? A mortality and hospitalisation follow-up for alcohol-related causes of the Finnish drinking habits surveys. Journal of Epidemiology & Community Health.

[bib22] Mäki N., Martikainen P., Eikemo T., Menvielle G., Lundberg O., Östergren O., Mackenbach J.P. (2013). Educational differences in disability-free life expectancy: A comparative study of long-standing activity limitation in eight European countries. Social Science & Medicine.

[bib23] Mäki N.E., Martikainen P.T., Eikemo T., Menvielle G., Lundberg O., Östergren O., Mackenbach J.P. (2014). The potential for reducing differences in life expectancy between educational groups in five European countries: The effects of obesity, physical inactivity and smoking. Journal of Epidemiology & Community Health.

[bib24] Marmot M.G. (2003). Understanding social inequalities in health. Perspectives in Biology and Medicine.

[bib25] Nusselder W.J., Looman C.W.N. (2004). Decomposition of differences in health expectancy by cause. Demography.

[bib26] Nusselder W.J., Looman C.W.N., Franco O.H., Peeters A., Slingerland A.S., Mackenbach J.P. (2008). The relation between non-occupational physical activity and years lived with and without disability. Journal of Epidemiology & Community Health.

[bib27] Pérez-Hernández B., Rubio-Valverde J.R., Nusselder W.J., Mackenbach J.P. (2019). Socioeconomic inequalities in disability in Europe: Contribution of behavioral, work-related and living conditions. The European Journal of Public Health.

[bib28] Rehm J., Kehoe T., Gmel G., Stinson F., Grant B., Gmel G. (2010). Statistical modeling of volume of alcohol exposure for epidemiological studies of population health: The US example. Population Health Metrics.

[bib29] Reuser M., Bonneux L.G., Willekens F.J. (2009). Smoking kills, obesity disables: A multistate approach of the US health and retirement survey. Obesity.

[bib30] Saito Y., Robine J.-M., Crimmins E.M. (2014). The methods and materials of health expectancy. Statistical Journal of the IAOS.

[bib31] Santiago C.D., Wadsworth M.E., Stump J. (2011). Socioeconomic status, neighborhood disadvantage, and poverty-related stress: Prospective effects on psychological syndromes among diverse low-income families. Journal of Economic Psychology.

[bib32] Schaap M.M., Kunst A.E., Leinsalu M., Regidor E., Ekholm O., Dzurova D., Mackenbach J.P. (2008). Effect of nationwide tobacco control policies on smoking cessation in high and low educated groups in 18 European countries. Tobacco Control.

[bib33] Siegel J.M., Yancey A.K., Aneshensel C.S., Schuler R. (1999). Body image, perceived pubertal timing, and adolescent mental health. Journal of Adolescent Health.

[bib34] Sullivan D.F. (1971). A single index of mortality and morbidity. HSMHA Health Reports.

[bib35] Van Oyen H., Bogaert P., Yokota R.T.C., Berger N. (2018). Measuring disability: A systematic review of the validity and reliability of the global activity limitations indicator (GALI). Archives of Public Health.

[bib36] Viester L., Verhagen E.A.L.M., Hengel K.M.O., Koppes L.L.J., van der Beek A.J., Bongers P.M. (2013). The relation between body mass index and musculoskeletal symptoms in the working population. BMC Musculoskeletal Disorders.

