# Supplementary material for: Determinants of educational inequalities in disability-free life expectancy between ages 35 and 80 in Europe
Source: SSM Popul Health. 2021 Jan 28;13:100740. doi: 10.1016/j.ssmph.2021.100740 (PMC7868628; doi:10.1016/j.ssmph.2021.100740)
Supplement: Multimedia component 1 [file mmc1.docx]

**Determinants of educational inequalities in disability-free life expectancy between ages 35 and 80 in Europe**

# Supplementary information

**Appendix table A1. Overview of data sources for mortality (age 35-80)**

| **Country** | **Design** | **Inclusion** | **Years** | **Person-years** | **Deaths** |
| --- | --- | --- | --- | --- | --- |
| Finland | Longitudinal | 80% sample | 2010-14 | 11644985 | 93850 |
| Sweden | Longitudinal | All | 2005-08 | 18238883 | 139884 |
| Norway | Longitudinal | All | 2006-09 | 9102575 | 86107 |
| Denmark | Longitudinal | All | 2010-14 | 15048315 | 127766 |
| England/Wales | Longitudinal | 1% sample | 2011-13 | 845053 | 5972 |
| Netherlands | Longitudinal |  | 2011-13 | 3736225 | 15612 |
| Belgium | Longitudinal | All | 2006-11 | 27184162 | 247709 |
| Austria | Longitudinal | All | 2010-14 | 9287635 | 67825 |
| Switzerland | Longitudinal | All nationals | 2011-13 | 12678229 | 81670 |
| France | Longitudinal | 1% sample | 2004-07 | 971147 | 7570 |
| Spain | Longitudinal | All | 2007-11 | 103180086 | 783281 |
| Hungary | Cross-sec. | All | 2010-12 | 16507050 | 232120 |
| Poland | Cross-sec. | All | 2010-12 | 56740803 | 658169 |
| Lithuania | Longitudinal | All | 2011-14 | 6273784 | 89212 |
| Estonia | Longitudinal | All | 2011-15 | 2751490 | 31450 |

**Appendix table B1. Overview of survey data**

|  | **European Social Survey (ESS)^a^** | | **EU-Statistics on Income and Living Conditions**  **(EU-SILC)^b^** | | | | |
| --- | --- | --- | --- | --- | --- | --- | --- |
| **Country** | **Analyses sample sizes** | **Individual Response Rate** | **Analyses sample sizes** | **Overall Personal Interview Response Rate**  **(1-*NRp)** | | | |
|  |  | **2014** |  | **2005*** | **2009*** | **2010** | **2014** |
| Finland | 1587 | 62,7 | 32869 | - | - | 82,3 | 80,1 |
| Sweden | 1300 | 50,1 | 17498 | na | 73,0 | - | - |
| Norway | 1058 | 53,9 | 14709 | na | 60,4 | - | - |
| Denmark | 1111 | 51,7 | 18175 | - | - | 51,7 | na |
| England/Wales | 1734 | 43,6 | 23784 | - | - | 70,2 | 61,3 |
| Netherlands | 1507 | 58,6 | 30577 | - | - | 80,7 | 82,0 |
| Belgium | 1261 | 57,0 | 17414 | - | - | 62,2 | 98,9 |
| Austria | 1408 | 51,6 | 17220 | - | - | 75,6 | 76,6 |
| Switzerland | 1135 | 52,7 | 11356 | - | - | 74,4 | Na |
| France | 1467 | 50,9 | 26226 | 83,0 | 78,1 | - | - |
| Spain | 1456 | 67,8 | 41924 | - | - | 80,5 | 77,7 |
| Hungary | 1343 | 52,7 | 29975 | - | - | 87,5 | 75,6 |
| Poland | 1186 | 65,8 | 42448 | - | - | 78,6 | 74,3 |
| Lithuania | 1765 | 68,9 | 16839 | - | - | 88,7 | 86,8 |
| Estonia | 1559 | 59,9 | 16772 | - | - | 79,8 | 70,8 |
|  |  |  |  |  |  |  |  |
| ^a^ Data for all risk factors with the exception of low income is obtained from the European Social Survey (ESS 2014). Global Activity Limitation Indicator (GALI) prevalences and low income estimates were obtained from EU-Statistics on Income and Living Conditions for individuals 35-80 years old.  ^b^ Three countries use pooled 2005 and 2009 EU-SILC waves for the estimation (Sweden, Norway, France) to better match with available mortality data. The rest of countries pool years 2010 and 2014 of EU-SILC  EU-SILC Quality reports obtained from GESIS German Microdata Lab site:  <https://www.gesis.org/en/missy/materials/EU-SILC/documents/quality-reports> | | | | | | | |

The European Social Survey (ESS) is a biennial cross-national survey starting from 2001. It surveys beliefs, attitudes and behavior patterns of populations of more than 30 countries. The samples are representative of all individuals over 15 years old living in private households and have a minimum size of 1.500 individuals, except for countries with less than 2 million inhabitants.

The European Union Statistics on Living Conditions (EU-SILC) survey provides annual data on variables on poverty, income, social exclusion and living conditions. The survey was launched in 2003, and has extended its coverage to the 28 member states of the enlarged European Union. The target population is all private households and their members living in the country’s territory. All household members are surveyed and only those above 16 years are interviewed. EU-SILC provides both cross-sectional and longitudinal data, intermediate years are excluded to avoid including subject more than once. We included 28 countries from EU-SILC, with a total sample size of 603,785. For EU-SILC, we pooled years 2010 and 2014, except for Sweden, Norway and France, where we used 2005 and 2009 to better match with the mortality data. Because EU-SILC is a rotating panel survey, we did not include intermediate years to avoid including respondents multiple times*.*

| \| **Appendix Table B2. Missings for Global Activity Limitation Indicator (GALI) and income from EU-SILC** \| \| \| \| \| \| \| \| \| \| \| \| --- \| --- \| --- \| --- \| --- \| --- \| --- \| --- \| --- \| --- \| --- \| \|  \| \|  \| **EU-Statistics on Income and Living Conditions** \| \| \| \| \| \| \| \| \| **(EU-SILC) ^ab^** \| \| \| \| \| \| \| \| \|  \| **Low Income** \| \| \| \| \| \| **Gali** \| \| \| \| \| **Country** \| \| **2005*** \| \| **2009*** \| **2010** \| **2014** \| **2005*** \| **2009*** \| **2010** \| **2014** \| \| Finland \| \|  \| \|  \| 47,4 \| 47,03 \| - \| - \| 48,5 \| 49,1 \| \| Sweden \| \| 47,6 \| \| 47,6 \|  \|  \| 48,4 \| 47,4 \| - \| - \| \| Norway \| \| 47,6 \| \| 47,6 \|  \|  \| 48,0 \| 46,9 \| - \| - \| \| Denmark \| \|  \| \|  \| 48,6 \| 47,96 \| - \| - \| 48,0 \| 47,8 \| \| England/Wales \| \|  \| \|  \| 0,5 \| 0,46 \| - \| - \| 1,4 \| 18,3 \| \| Netherlands \| \|  \| \|  \| 44,3 \| 44,52 \| - \| - \| 44,2 \| 44,4 \| \| Belgium \| \|  \| \|  \| 0,1 \| 0,15 \| - \| - \| 1,2 \| 0,9 \| \| Austria \| \|  \| \|  \| 0 \| 0,02 \| - \| - \| 0,1 \| 0,0 \| \| Switzerland \| \|  \| \|  \| 10,9 \| - \| - \| - \| 10,9 \| NA \| \| France \| \| 0,6 \| \| 0,98 \|  \|  \| 0,1 \| 0.5 \| - \| - \| \| Spain \| \|  \| \|  \| 1,8 \| 1,11 \| - \| - \| 1,2 \| 0,5 \| \| Hungary \| \|  \| \|  \| 0,0 \| 0,0 \| - \| - \| 1,1 \| 0,3 \| \| Poland \| \|  \| \|  \| 5,8 \| 0,1 \| - \| - \| 5,8 \| 5,9 \| \| Lithuania \| \|  \| \|  \| 0,3 \| 0,2 \| - \| - \| 12,1 \| 14,9 \| \| Estonia \| \|  \| \|  \| 0,2 \| 0,6 \| - \| - \| 18,8 \| 24,1 \| \|  \| \|  \| \|  \| - \| - \|  \|  \|  \|  \| \| ^a^ Data for all risk factors with the exception of low income is obtained from the European Social Survey (ESS 2014). Global Activity Limitation Indicator (GALI) prevalences and low income estimates were obtained from EU-Statistics on Income and Living Conditions for individuals 35-80 years old. \| \| \| \| \| \| \| \| \| \| \| ^b^ Three countries use pooled 2005 and 2009 EU-SILC waves for the estimation (Sweden, Norway, France) to better match with available mortality data. The rest of countries pool years 2010 and 2014 of EU-SILC \| \| \| \| \| \| \| \| \| \| \| EU-SILC Quality reports obtained from GESIS German Microdata Lab site: \| \| \| \| \| \| \| \| \| \| \| <https://www.gesis.org/en/missy/materials/EU-SILC/documents/quality-reports> \| \| \| \| \| \| \| \| \| \|  \| **Appendix Table B3. Missings for risk factors from European Social Survey (ESS) - %** \| \| \| \| \| \| \| \| \| --- \| --- \| --- \| --- \| --- \| --- \| --- \| --- \| \| Country \| High body-weight \| Smoking \| Low fruit & veg \| Fathers manual occupation \| Low Physical Activity \| Few social contacts \| High alcohol consumption \| \| Austria \| 3,0 \| 0,1 \| 0,1 \| 12,4 \| 0,0 \| 0,1 \| 1,1 \| \| Belgium \| 0,3 \| 0,1 \| 0,0 \| 10,6 \| 0,0 \| 0,0 \| 0,0 \| \| Switzerland \| 1,4 \| 0,1 \| 0,1 \| 8,6 \| 0,0 \| 0,1 \| 0,9 \| \| Denmark \| 2,1 \| 0,3 \| 0,2 \| 7,3 \| 0,0 \| 0,3 \| 8,2 \| \| Estonia \| 2,0 \| 0,1 \| 0,1 \| 22,8 \| 0,0 \| 0,2 \| 1,8 \| \| Spain \| 4,1 \| 0,1 \| 0,2 \| 10,5 \| 0,0 \| 0,2 \| 0,4 \| \| Finland \| 2,4 \| 0,1 \| 0,1 \| 13,7 \| 0,0 \| 0,1 \| 0,9 \| \| France \| 1,8 \| 0,0 \| 0,2 \| 14,6 \| 0,0 \| 0,1 \| 0,5 \| \| Hungary \| 2,4 \| 0,1 \| 0,0 \| 13,3 \| 0,0 \| 0,2 \| 0,5 \| \| Lithuania \| 5,6 \| 0,4 \| 1,1 \| 29,1 \| 0,0 \| 2,8 \| 6,1 \| \| Netherlands \| 0,1 \| 0,1 \| 0,0 \| 8,9 \| 0,0 \| 0,0 \| 0,1 \| \| Norway \| 2,2 \| 0,2 \| 0,1 \| 7,3 \| 0,0 \| 0,0 \| 0,7 \| \| Poland \| 2,0 \| 0,0 \| 0,2 \| 14,6 \| 0,0 \| 1,2 \| 2,1 \| \| Sweden \| 0,9 \| 0,3 \| 0,3 \| 9,7 \| 0,0 \| 0,2 \| 1,1 \| \| England& Wales \| 5,7 \| 0,0 \| 0,1 \| 16,3 \| 0,0 \| 0,1 \| 0,2 \| |
| --- | --- | --- | --- | --- | --- | --- | --- | --- | --- | --- | --- | --- | --- | --- | --- | --- | --- | --- | --- | --- | --- | --- | --- | --- | --- | --- | --- | --- | --- | --- | --- | --- | --- | --- | --- | --- | --- | --- | --- | --- | --- | --- | --- | --- | --- | --- | --- | --- | --- | --- | --- | --- | --- | --- | --- | --- | --- | --- | --- | --- | --- | --- | --- | --- | --- | --- | --- | --- | --- | --- | --- | --- | --- | --- | --- | --- | --- | --- | --- | --- | --- | --- | --- | --- | --- | --- | --- | --- | --- | --- | --- | --- | --- | --- | --- | --- | --- | --- | --- | --- | --- | --- | --- | --- | --- | --- | --- | --- | --- | --- | --- | --- | --- | --- | --- | --- | --- | --- | --- | --- | --- | --- | --- | --- | --- | --- | --- | --- | --- | --- | --- | --- | --- | --- | --- | --- | --- | --- | --- | --- | --- | --- | --- | --- | --- | --- | --- | --- | --- | --- | --- | --- | --- | --- | --- | --- | --- | --- | --- | --- | --- | --- | --- | --- | --- | --- | --- | --- | --- | --- | --- | --- | --- | --- | --- | --- | --- | --- | --- | --- | --- | --- | --- | --- | --- | --- | --- | --- | --- | --- | --- | --- | --- | --- | --- | --- | --- | --- | --- | --- | --- | --- | --- | --- | --- | --- | --- | --- | --- | --- | --- | --- | --- | --- | --- | --- | --- | --- | --- | --- | --- | --- | --- | --- | --- | --- | --- | --- | --- | --- | --- | --- | --- | --- | --- | --- | --- | --- | --- | --- | --- | --- | --- | --- | --- | --- | --- | --- | --- | --- | --- | --- | --- | --- | --- | --- | --- | --- | --- | --- | --- | --- | --- | --- | --- | --- | --- | --- | --- | --- | --- | --- | --- | --- | --- | --- | --- | --- | --- | --- | --- | --- | --- | --- | --- | --- | --- | --- | --- | --- | --- | --- | --- | --- | --- | --- | --- | --- | --- | --- | --- | --- | --- | --- | --- | --- | --- | --- | --- | --- | --- | --- | --- | --- | --- | --- | --- | --- | --- | --- | --- | --- | --- | --- | --- | --- | --- | --- | --- | --- | --- | --- | --- | --- | --- | --- | --- | --- | --- | --- | --- | --- | --- | --- | --- | --- | --- | --- | --- | --- | --- | --- | --- | --- | --- | --- | --- | --- | --- | --- | --- | --- | --- | --- | --- | --- | --- | --- | --- | --- | --- | --- | --- | --- | --- | --- | --- | --- | --- | --- | --- | --- | --- | --- | --- | --- | --- | --- | --- | --- | --- | --- | --- | --- | --- | --- | --- | --- | --- | --- | --- | --- | --- | --- |

**Appendix table C1. Survey questions for risk factors**

| **Source** | **Risk factor** | **Question** | **Categories** | **Variable** |
| --- | --- | --- | --- | --- |
| ESS | Manual Occupation of the father | Which of the descriptions on this card best describes the sort of work he [father] did when you were 14? | 1.Professional and technical occupations (doctor, teacher, engineer, artist, accountant)  2. Higher administrator occupations (i.e. banker, executive in big business, high government  3. Clerical occupations ( i.e. secretary, clerk, office manager, book keeper)  4. Sales occupations (i.e. sales manager, shop owner, shop assistant, insurance agent)  5. Service occupations (restaurant owner, police officer, waiter, caretaker, barber, armed forces)  6. Skilled worker (i.e. foreman, motor mechanic, printer, tool and die maker, electrician) | Non-manual (Reference) |
|  |  |  | 7. Semi-skilled worker (bricklayer, bus driver, cannery worker, carpenter, sheet metal worker, baker)  8, Unskilled worker (labourer, porter, unskilled factory worker)  9. Farm worker (i.e. farmer, farm labourer, tractor driver, fisherman) | Manual |
| EU-SILC | Lowest Inc. Quint. | Household equivalized income converted into quintiles | Lowest quintile | Low income |
|  |  |  | Fourth, third, second, highest quintiles | Not low income (Reference) |
| ESS | Social contact | Using this card, how often do you meet socially (by choice rather than work or duty) with friends, relatives or work colleagues | Never  Less than once a month  several times a month | Low social contact |
|  |  |  | Once a week  Several times a week, everyday | Not low social contact (Reference) |
| ESS | Smoking | Now thinking about smoking cigarettes. Which of the descriptions on this card best describes your smoking behaviour? Include rolled tobacco, but not pipes, cigars, or electronic cigarettes | Smoke daily  Smoke but not everyday | Current smoker |
|  |  |  | I don’t smoke now but I used to | Former smoker |
|  |  |  | I have never smoked  ,I have only smoked a few times | Never smokier (Reference) |
| ESS | Alcohol (uses information on three questions to derive avg g/day and use this as measure of alcohol consumption) | In the last 12 months, that is since [MONTH, YEAR], how often have you had a drink containing alcohol? This could be wine, beer, cider, spirits or other drinks containing alcohol. Please choose an answer from this card.  Please think about the last time you were drinking alcohol on a Monday, a Tuesday, a Wednesday or a Thursday. How many of each of the following drinks did you have on that day?  Now please think about the last time you were drinking alcohol on a Friday, a Saturday or a Sunday. How many of each of the following drinks did you have on that day? | Everyday  several times a week  2-3 times a month  less than once a month,  never  refused, don’t know  Number of drinks per category  Number of drinks per category | Alcohol /<25g avg daily (Reference) |
|  |  |  |  | Alcohol 25-44.9 avg g/day |
|  |  |  |  | Alcohol >45 avg g/day |
| ESS | BMI | Height and weight variables | cm/kg | Normal (BMI <25 kg/m^2^)  Overweight BMI25-30 kg/m^2^)  Obese (BMI >30) |
| ESS | Physical Activity | On how many of the last 7 days did you walk quickly, do sports or other physical activity for 30 minutes or longer? | 0-7 | Low physical activity: (<5), no risk (>=5) |
|  |  |  |  | Not low physical activity: >5 (Reference) |
| ESS | Fruit & Vegetable (uses information on two questions for daily fruit and vegetable consumption) | Using this card, please tell me how often you eat fruit, excluding drinking juice?  Using the same card, please tell me how often you eat vegetables or salad, excluding potatoes? | Three times or more a day  twice a day  once a day  less than once a day but at least 4 times a week  less than 4 times a week but at least once a week  less than once a week  never  don’t know | At least once a day (Reference)  Fruit and vegetable |
|  |  |  |  | Low: not at least once a day  Fruit and vegetable |

**Appendix Table D1. Overview of Relative Risks for mortality and disability**

**Overview of Relative Risks for mortality**

| Father's occupation | All other (=ref) | 1.0 | |  |
| --- | --- | --- | --- | --- |
|  | Manual class | 1.1 | | [[1](#_ENREF_1), [9-18](#_ENREF_9)] |
| Income | All other (=ref) | 1.0 | |  |
|  | Lowest quintile | 1.3 | | [[19](#_ENREF_19)] |
| Social contact | All other (=ref) | 1.0 | |  |
|  | less than once a week | 1.1 | | [[20](#_ENREF_20)] |
| Smoking | Never (=ref) | 1.0 | |  |
|  | Former | 1.3 | | [[21](#_ENREF_21)] |
|  | Current | 2.2 | | [[21](#_ENREF_21)] |
| Alcohol consumption | average gr/day <24.9 (=ref) | 1.0 | |  |
|  | average gr/day = 25-44.9 | 1.1 | | [[22](#_ENREF_22)] |
|  | average gr/day >=45 | 1.4 | | [[22](#_ENREF_22)] |
| Overweight/obesity | Normal (BMI<25)(=ref) | 1.0 | |  |
|  | Overweight (BMI=25-29) | 1.1 | | [[23](#_ENREF_23)] |
|  | Obese (BMI>=30) | 1.7 | | [[23](#_ENREF_23)] |
| Physical activity | At least 5 days per week (=ref) | 1.0 | |  |
|  | Less than 5 days per week | 1.3 | | [[21](#_ENREF_21)] |
| Fruit and vegetable consumption | At least once a day (=ref) | 1.0 | |  |
|  | Less than once a day | | 1.2 | [[24](#_ENREF_24)] |

*Where needed categories in the sources were pooled to reflect the exposure categories

|  |
| --- |

Relative risks were preferably based on meta-analyses or reviews. If these were not available we used individual studies. Where needed, we converted the relative risks for the exposure categories in the respective papers into the categories in our study that match with the exposure categories based on the ESS survey. The RR are adjusted for different sets of confounders, depending on the study. If more Relative Risks were presented in the literature, we selected Relative Risks adjusted for age and gender and other risk factors not on the causal pathway between the risk factor and mortality/disability, and for adult socioeconomic position.

If more Relative Risks were presented in the literature, we selected Relative Risks adjusted for age and gender and other risk factors not on the causal pathway between the risk factor and mortality/disability, and for adult socioeconomic position. We averaged the selected relative risk for obtain the relative risk. We converted the RR into a beta by using ln(RR), averaged the beta’s, then converted the average beta into RR by using exp(beta). If the RR were separate for men and women, we first calculated average, following the same procedure.

| **Appendix Table D2. Overview of Relative Risks for disability**   \| Risk Factor \| Level \| Main Analysis \| \| Source* \|  \| \| --- \| --- \| --- \| --- \| --- \| --- \| \| Father's occupation \| All other (=ref) \| 1 \| \|  \| \|  \| Manual class \| 1.3 \| \| [[25-27](#_ENREF_25)] \|  \| \| Income \| All other (=ref) \| 1 \| \|  \|  \| \|  \| Lowest quintile \| 1.5 \| \| [[28-33](#_ENREF_28)] \|  \| \| Social contact \| All other (=ref) \| 1 \| \|  \|  \| \|  \| less than once a week \| 1.05 \| \| Assumed same as for mortality \|  \| \| Smoking \| Never (=ref) \| 1 \| \|  \|  \| \|  \| Former \| 1.1 \| \|  \| \|  \| Current \| 1.3 \| \|  \| \| Alcohol consumption \| average gr/day <24.9 (=ref) \| 1 \| \|  \| \|  \| average gr/day = 25-44.9 \| 1 \| \| [[34-37](#_ENREF_34)] \|  \| \|  \| average gr/day >=45 \| 1.05 \| \| [[34-37](#_ENREF_34)] \|  \| \| Body weight \| Normal (BMI<25)(=ref) \| 1 \| \|  \| \|  \| Overweight (BMI=25-29) \| 1.4 \| \| [[36-40](#_ENREF_36)] \| \|  \| Obese (BMI>=30) \| 1.8 \| \|  \|  \| \| Physical activity \| At least 5 days per week (=ref) \| 1 \| \|  \|  \| \|  \| Less than 5 days per week \| 1.5 \| \| [[41](#_ENREF_41)] \| \| Fruit and vegetable consumption \| At least once a day (=ref) \| 1 \| \|  \| \|  \| Less than once a day \| \| 1.2 \| [[34](#_ENREF_34)] \|   *Where needed categories in the sources were pooled to reflect the exposure categories | |
| --- | --- | --- | --- | --- | --- | --- | --- | --- | --- | --- | --- | --- | --- | --- | --- | --- | --- | --- | --- | --- | --- | --- | --- | --- | --- | --- | --- | --- | --- | --- | --- | --- | --- | --- | --- | --- | --- | --- | --- | --- | --- | --- | --- | --- | --- | --- | --- | --- | --- | --- | --- | --- | --- | --- | --- | --- | --- | --- | --- | --- | --- | --- | --- | --- | --- | --- | --- | --- | --- | --- | --- | --- | --- | --- | --- | --- | --- | --- | --- | --- | --- | --- | --- | --- | --- | --- | --- | --- | --- | --- | --- | --- | --- | --- | --- | --- | --- | --- | --- | --- | --- | --- | --- | --- | --- | --- | --- | --- | --- | --- | --- | --- |
|  | |

Relative risks were preferably based on reviews. If these were not available we used individual studies. Where needed, we converted the relative risks for the exposure categories in the respective papers into the categories in our study that match with the exposure categories based on the ESS survey. The RR are adjusted for different sets of confounders, depending on the study. If more Relative Risks were presented in the literature, we selected Relative Risks adjusted for age and gender and other risk factors not on the causal pathway between the risk factor and mortality/disability, and for adult socioeconomic position.

If more Relative Risks were presented in the literature, we selected Relative Risks adjusted for age and gender and other risk factors not on the causal pathway between the risk factor and mortality/disability, and for adult socioeconomic position. We averaged the selected relative risk for obtain the relative risk. We converted the RR into a beta by using ln(RR), averaged the beta’s, then converted the average beta into RR by using exp(beta). If the RR were separate for men and women, we first calculated average, following the same procedure.

**References:**

1 Galobardes B, Lynch JW, Smith GD. Is the association between childhood socioeconomic circumstances and cause-specific mortality established? Update of a systematic review. *J Epidemiol Community Health* 2008;**62**:387-90.

2 Aldabe B, Anderson R, Lyly-Yrjanainen M*, et al.* Contribution of material, occupational, and psychosocial factors in the explanation of social inequalities in health in 28 countries in Europe. *J Epidemiol Community Health* 2011;**65**:1123-31.

3 Standfeld SA. Social support and social cohesion. In: Marmot M, Wilkinson RG, eds. *Social Determinants of health*. Oxford: Osford University Press 2006:148-71.

4 Hiscock R, Bauld L, Amos A*, et al.* Socioeconomic status and smoking: a review. *Ann N Y Acad Sci* 2012;**1248**:107-23.

5 Devaux M, Sassi F. Alcohol consumption and harmful drinking: trends and social disparities across OECD countries. Paris: OECD 2015.

6 Roskam AJ, Kunst AE, Van Oyen H*, et al.* Comparative appraisal of educational inequalities in overweight and obesity among adults in 19 European countries. *Int J Epidemiol* 2010;**39**:392-404.

7 Beenackers MA, Kamphuis CB, Giskes K*, et al.* Socioeconomic inequalities in occupational, leisure-time, and transport related physical activity among European adults: a systematic review. *Int J Behav Nutr Phys Act* 2012;**9**:116.

8 Irala-Estevez JD, Groth M, Johansson L*, et al.* A systematic review of socio-economic differences in food habits in Europe: consumption of fruit and vegetables. *Eur J Clin Nutr* 2000;**54**:706-14.

9 Elo IT, Martikainen P, Myrskyla M. Socioeconomic status across the life course and all-cause and cause-specific mortality in Finland. *Soc Sci Med* 2014;**119**:198-206.

10 Hayward MD, Gorman BK. The long arm of childhood: the influence of early-life social conditions on men's mortality. *Demography* 2004;**41**:87-107.

11 Juarez SP, Goodman A, Koupil I. From cradle to grave: tracking socioeconomic inequalities in mortality in a cohort of 11 868 men and women born in Uppsala, Sweden, 1915-1929. *J Epidemiol Commun H* 2016;**70**:569-75.

12 Kelly-Irving M, Lepage B, Dedieu D*, et al.* Adverse childhood experiences and premature all-cause mortality. *Eur J Epidemiol* 2013;**28**:721-34.

13 Khang YH. Relationship between childhood socio-economic position and mortality risk in adult males of the Korea Labour and Income Panel Study (KLIPS). *Public Health* 2006;**120**:724-31.

14 Lawlor DA, Sterne JA, Tynelius P*, et al.* Association of childhood socioeconomic position with cause-specific mortality in a prospective record linkage study of 1,839,384 individuals. *Am J Epidemiol* 2006;**164**:907-15.

15 Power C, Hypponen E, Smith GD. Socioeconomic position in childhood and early adult life and risk of mortality: a prospective study of the mothers of the 1958 British birth cohort. *Am J Public Health* 2005;**95**:1396-402.

16 Strand BH, Kunst A. Childhood socioeconomic position and cause-specific mortality in early adulthood. *Am J Epidemiol* 2007;**165**:85-93.

17 Stringhini S, Dugravot A, Kivimaki M*, et al.* Do different measures of early life socioeconomic circumstances predict adult mortality? Evidence from the British Whitehall II and French GAZEL studies. *J Epidemiol Commun H* 2011;**65**:1097-103.

18 Galobardes B, Lynch JW, Davey Smith G. Childhood socioeconomic circumstances and cause-specific mortality in adulthood: systematic review and interpretation. *Epidemiol Rev* 2004;**26**:7-21.

19 Martikainen P, Makela P, Koskinen S*, et al.* Income differences in mortality: a register-based follow-up study of three million men and women. *Int J Epidemiol* 2001;**30**:1397-405.

20 Shor E, Roelfs DJ. Social contact frequency and all-cause mortality: a meta-analysis and meta-regression. *Soc Sci Med* 2015;**128**:76-86.

21 Stringhini S, Carmeli C, Jokela M*, et al.* Socioeconomic status and the 25 x 25 risk factors as determinants of premature mortality: a multicohort study and meta-analysis of 1.7 million men and women. *Lancet* 2017;**389**:1229-37.

22 Stockwell T, Zhao J, Panwar S*, et al.* Do "Moderate" Drinkers Have Reduced Mortality Risk? A Systematic Review and Meta-Analysis of Alcohol Consumption and All-Cause Mortality. *J Stud Alcohol Drugs* 2016;**77**:185-98.

23 Di Angelantonio E, Bhupathiraju SN, Wormser D*, et al.* Body-mass index and all-cause mortality: individual-participant-data meta-analysis of 239 prospective studies in four continents. *Lancet* 2016;**388**:776-86.

24 Wang X, Ouyang Y, Liu J*, et al.* Fruit and vegetable consumption and mortality from all causes, cardiovascular disease, and cancer: systematic review and dose-response meta-analysis of prospective cohort studies. *BMJ* 2014;**349**:g4490.

25 Guralnik JM, Butterworth S, Wadsworth ME*, et al.* Childhood socioeconomic status predicts physical functioning a half century later. *J Gerontol A Biol Sci Med Sci* 2006;**61**:694-701.

26 Montez JK, Hayward MD. Cumulative childhood adversity, educational attainment, and active life expectancy among U.S. adults. *Demography* 2014;**51**:413-35.

27 Osler M, Madsen M, Nybo Andersen AM*, et al.* Do childhood and adult socioeconomic circumstances influence health and physical function in middle-age? *Soc Sci Med* 2009;**68**:1425-31.

28 Broese van Groenou MI, Deeg DJ, Penninx BW. Income differentials in functional disability in old age: relative risks of onset, recovery, decline, attrition and mortality. *Aging Clin Exp Res* 2003;**15**:174-83.

29 Cambois E, Sole-Auro A, Robine JM. Economic Hardship and Educational Differentials in Disability in 26 European Countries. *J Aging Health* 2016;**28**:1214-38.

30 Fuller-Thomson E, Gadalla T. Income inequality and limitations in activities of daily living: a multilevel analysis of the 2003 American Community Survey. *Public Health* 2008;**122**:221-8.

31 Nordstrom CK, Diez Roux AV, Schulz R*, et al.* Socioeconomic position and incident mobility impairment in the Cardiovascular Health Study. *BMC Geriatr* 2007;**7**:11.

32 Rueda S, Artazcoz L, Navarro V. Health inequalities among the elderly in western Europe. *J Epidemiol Community Health* 2008;**62**:492-8.

33 von dem Knesebeck O, Luschen G, Cockerham WC*, et al.* Socioeconomic status and health among the aged in the United States and Germany: a comparative cross-sectional study. *Soc Sci Med* 2003;**57**:1643-52.

34 Artaud F, Dugravot A, Sabia S*, et al.* Unhealthy behaviours and disability in older adults: three-City Dijon cohort study. *BMJ* 2013;**347**:f4240.

35 Kim LG, Adamson J, Ebrahim S. Influence of life-style choices on locomotor disability, arthritis and cardiovascular disease in older women: prospective cohort study. *Age Ageing* 2013;**42**:696-701.

36 Ostbye T, Taylor DH, Jr., Krause KM*, et al.* The role of smoking and other modifiable lifestyle risk factors in maintaining and restoring lower body mobility in middle-aged and older Americans: results from the HRS and AHEAD. Health and Retirement Study. Asset and Health Dynamics Among the Oldest Old. *J Am Geriatr Soc* 2002;**50**:691-9.

37 Tas U, Verhagen AP, Bierma-Zeinstra SM*, et al.* Incidence and risk factors of disability in the elderly: the Rotterdam Study. *Prev Med* 2007;**44**:272-8.

38 Walter S, Kunst A, Mackenbach J*, et al.* Mortality and disability: the effect of overweight and obesity. *Int J Obes (Lond)* 2009;**33**:1410-8.

39 Zhang S, Tomata Y, Sugiyama K*, et al.* Body mass index and the risk of incident functional disability in elderly Japanese The OHSAKI Cohort 2006 Study. *Medicine* 2016;**95**.

40 He XXZ, Baker DW. Body mass index, physical activity, and the risk of decline in overall health and physical functioning in late middle age. *Am J Public Health* 2004;**94**:1567-73.

41 Tak E, Kuiper R, Chorus A*, et al.* Prevention of onset and progression of basic ADL disability by physical activity in community dwelling older adults: a meta-analysis. *Ageing Res Rev* 2013;**12**:329-38.

|  | | | | | | | | | | |  |  |
| --- | --- | --- | --- | --- | --- | --- | --- | --- | --- | --- | --- | --- |
|  | **Male** | | | | | | | | | | | |
| **Country** | **Low** | **95% CI** | | **Mid** | **95% CI** | | **High** | **95% CI** | | **Gap** | **95% CI** | |
| Finland | 23.5 | 22.6 | 24.5 | 26.4 | 25.7 | 27.1 | 31.7 | 30.9 | 32.3 | 8.1 | 7.0 | 9.3 |
| Sweden | 29.7 | 28.5 | 30.6 | 32.5 | 32.0 | 33.2 | 36.9 | 35.7 | 37.5 | 7.2 | 5.7 | 8.3 |
| Norway | 27.5 | 26.5 | 28.8 | 32.3 | 31.6 | 32.9 | 38.0 | 37.4 | 38.6 | 10.4 | 9.1 | 11.7 |
| Denmark | 26.1 | 24.5 | 27.0 | 29.3 | 28.5 | 30.1 | 32.3 | 31.2 | 33.2 | 6.2 | 4.8 | 7.9 |
| England/Wales | 26.4 | 25.8 | 27.0 | 31.8 | 31.0 | 32.4 | 34.6 | 34.1 | 35.3 | 8.2 | 7.5 | 9.2 |
| Netherlands | 26.3 | 25.3 | 27.4 | 29.7 | 29.0 | 30.5 | 33.1 | 32.2 | 33.8 | 6.8 | 5.3 | 8.2 |
| Belgium | 24.9 | 24.1 | 25.7 | 31.0 | 30.3 | 31.6 | 34.9 | 34.1 | 35.3 | 10.0 | 8.9 | 10.8 |
| Austria | 19.2 | 18.0 | 20.4 | 25.8 | 25.3 | 26.3 | 30.9 | 30.2 | 31.5 | 11.7 | 10.3 | 13.1 |
| Switzerland | 25.7 | 23.8 | 27.6 | 30.8 | 30.1 | 31.5 | 34.2 | 33.5 | 34.8 | 8.5 | 6.3 | 10.5 |
| France | 26.4 | 25.9 | 27.0 | 30.2 | 29.6 | 30.6 | 35.0 | 34.4 | 35.8 | 8.6 | 7.8 | 9.6 |
| Spain | 28.1 | 27.9 | 28.3 | 31.4 | 30.9 | 32.1 | 34.3 | 33.3 | 34.4 | 6.2 | 5.2 | 7.4 |
| Hungary | 20.1 | 19.4 | 20.7 | 26.4 | 26.1 | 26.8 | 31.5 | 30.9 | 32.2 | 11.5 | 10.6 | 12.4 |
| Poland | 22.0 | 21.1 | 22.7 | 27.4 | 27.0 | 27.7 | 32.6 | 32.0 | 33.6 | 10.7 | 9.8 | 12.0 |
| Lithuania | 19.3 | 17.7 | 20.9 | 26.1 | 25.6 | 26.6 | 32.5 | 31.8 | 33.5 | 13.2 | 11.5 | 15.2 |
| Estonia | 16.6 | 15.6 | 17.8 | 22.7 | 22.1 | 23.3 | 27.6 | 26.8 | 28.5 | 11.0 | 9.5 | 12.2 |
| **Average** | 25.4 | 25.2 | 25.7 | 30.0 | 29.8 | 30.2 | 33.9 | 33.6 | 34.1 | 8.5 | 8.1 | 8.8 |
|  |  |  |  |  |  |  |  |  |  |  |  |  |
|  | **Female** | | | | | | | | | | | |
| **Country** | **Low** | **95% CI** | | **Mid** | **95% CI** | | **High** | **95% CI** | | **Gap** | **95% CI** | |
| Finland | 24.4 | 23.1 | 25.5 | 25.8 | 25.1 | 26.8 | 29.3 | 28.6 | 30.0 | 4.9 | 3.6 | 6.5 |
| Sweden | 27.1 | 25.6 | 29.1 | 30.8 | 30.1 | 31.4 | 34.0 | 33.2 | 35.2 | 6.9 | 5.1 | 8.8 |
| Norway | 25.8 | 24.6 | 27.2 | 31.9 | 31.1 | 32.6 | 35.3 | 34.4 | 36.2 | 9.4 | 7.8 | 11.0 |
| Denmark | 26.2 | 24.5 | 27.4 | 28.8 | 27.8 | 29.5 | 30.0 | 29.0 | 31.0 | 3.8 | 2.4 | 5.7 |
| England/Wales | 26.7 | 26.0 | 27.3 | 31.9 | 31.3 | 32.5 | 33.5 | 33.0 | 34.4 | 6.9 | 6.1 | 7.9 |
| Netherlands | 23.8 | 22.7 | 24.7 | 27.3 | 26.3 | 28.0 | 31.3 | 30.4 | 32.1 | 7.4 | 6.1 | 8.8 |
| Belgium | 24.3 | 23.5 | 25.0 | 29.9 | 29.2 | 30.5 | 33.6 | 32.9 | 34.6 | 9.3 | 8.4 | 10.5 |
| Austria | 22.2 | 21.3 | 23.0 | 27.6 | 27.0 | 28.1 | 30.3 | 29.3 | 31.3 | 8.1 | 6.9 | 9.6 |
| Switzerland | 28.2 | 26.9 | 29.3 | 30.9 | 30.1 | 31.5 | 30.7 | 29.6 | 32.0 | 2.6 | 0.9 | 4.4 |
| France | 27.8 | 27.3 | 28.2 | 31.5 | 30.8 | 32.0 | 34.6 | 33.2 | 35.2 | 6.7 | 5.4 | 7.5 |
| Spain | 27.9 | 27.7 | 28.2 | 32.8 | 31.9 | 33.3 | 34.9 | 34.4 | 35.6 | 6.9 | 6.4 | 7.7 |
| Hungary | 20.2 | 19.6 | 20.9 | 28.0 | 27.6 | 28.4 | 32.4 | 31.8 | 33.2 | 12.2 | 11.3 | 13.1 |
| Poland | 24.6 | 24.0 | 25.5 | 28.7 | 28.3 | 29.0 | 32.3 | 31.7 | 33.2 | 7.8 | 6.7 | 8.7 |
| Lithuania | 21.2 | 19.3 | 23.9 | 27.6 | 27.0 | 28.2 | 34.5 | 33.9 | 35.4 | 13.3 | 10.5 | 15.3 |
| Estonia | 18.0 | 16.8 | 20.0 | 23.8 | 23.2 | 24.4 | 29.7 | 28.9 | 30.3 | 11.7 | 9.7 | 12.9 |
| **Average** | 26.0 | 25.8 | 26.2 | 30.5 | 30.2 | 30.7 | 33.2 | 33.0 | 33.5 | 7.2 | 6.9 | 7.6 |

**Appendix Table E1. Educational Inequalities in disability-free life expectancy, by gender and country**

**Appendix Table E2. Educational Inequalities in life expectancy, by gender and country**

|  | | | | | | | | | | |  |  |
| --- | --- | --- | --- | --- | --- | --- | --- | --- | --- | --- | --- | --- |
|  | **Male** | | | | | | | | | | | |
| **Country** | **Low** | **95% CI** | | **Mid** | **95% CI** | | **High** | **95% CI** | | **Gap** | **95% CI** | |
| Finland | 37,4 | 37,3 | 37,5 | 39,3 | 39,3 | 39,4 | 41,5 | 41,5 | 41,6 | 4,1 | 4,0 | 4,2 |
| Sweden | 39,3 | 39,2 | 39,3 | 40,5 | 40,4 | 40,5 | 41,9 | 41,8 | 41,9 | 2,6 | 2,5 | 2,7 |
| Norway | 37,9 | 37,5 | 38,3 | 40,2 | 40,0 | 40,4 | 41,8 | 41,5 | 42,0 | 3,9 | 3,4 | 4,3 |
| Denmark | 37,5 | 37,4 | 37,6 | 40,0 | 40,0 | 40,1 | 41,7 | 41,7 | 41,8 | 4,2 | 4,1 | 4,3 |
| England/Wales | 39,4 | 39,1 | 39,6 | 41,5 | 41,2 | 41,7 | 42,1 | 41,9 | 42,3 | 2,7 | 2,4 | 3,0 |
| Netherlands | 39,7 | 39,6 | 39,8 | 40,9 | 40,8 | 41,1 | 42,0 | 41,9 | 42,1 | 2,3 | 2,2 | 2,6 |
| Belgium | 38,5 | 38,4 | 38,5 | 39,8 | 39,8 | 39,9 | 41,2 | 41,1 | 41,2 | 2,7 | 2,6 | 2,8 |
| Austria | 38,3 | 38,1 | 38,4 | 39,8 | 39,8 | 39,9 | 41,8 | 41,7 | 41,9 | 3,5 | 3,4 | 3,6 |
| Switzerland | 39,1 | 39,0 | 39,2 | 41,0 | 41,0 | 41,0 | 42,3 | 42,2 | 42,3 | 3,2 | 3,0 | 3,3 |
| France | 37,6 | 37,3 | 37,9 | 39,5 | 39,3 | 39,7 | 41,3 | 41,1 | 41,7 | 3,7 | 3,3 | 4,1 |
| Spain | 39,2 | 39,2 | 39,3 | 40,4 | 40,4 | 40,4 | 41,3 | 41,3 | 41,3 | 2,1 | 2,0 | 2,1 |
| Hungary | 33,8 | 33,8 | 33,9 | 38,3 | 38,2 | 38,4 | 40,1 | 40,0 | 40,2 | 6,3 | 6,2 | 6,4 |
| Poland | 34,2 | 34,1 | 34,2 | 38,2 | 38,2 | 38,2 | 40,7 | 40,6 | 40,7 | 6,5 | 6,5 | 6,5 |
| Lithuania | 31,2 | 31,0 | 31,4 | 35,2 | 35,1 | 35,3 | 39,4 | 39,3 | 39,6 | 8,2 | 8,0 | 8,4 |
| Estonia | 32,8 | 32,6 | 33,1 | 36,6 | 36,5 | 36,7 | 40,1 | 40,0 | 40,3 | 7,3 | 7,0 | 7,6 |
| **Average** | 37,8 | 37,6 | 37,9 | 39,9 | 39,8 | 40,0 | 41,3 | 41,2 | 41,5 | 3,6 | 3,4 | 3,7 |
|  |  |  |  |  |  |  |  |  |  |  |  |  |
|  | **Female** | | | | | | | | | | | |
| **Country** | **Low** | **95% CI** | | **Mid** | **95% CI** | | **High** | **95% CI** | | **Gap** | **95% CI** | |
| Finland | 40,6 | 40,5 | 40,7 | 42,2 | 42,2 | 42,3 | 43,0 | 43,0 | 43,1 | 2,4 | 2,3 | 2,5 |
| Sweden | 41,1 | 41,0 | 41,1 | 42,0 | 42,0 | 42,1 | 42,8 | 42,8 | 42,9 | 1,8 | 1,7 | 1,8 |
| Norway | 40,6 | 40,4 | 40,8 | 42,1 | 42,0 | 42,2 | 42,9 | 42,7 | 43,0 | 2,2 | 1,9 | 2,5 |
| Denmark | 40,0 | 39,9 | 40,0 | 41,8 | 41,8 | 41,9 | 42,6 | 42,6 | 42,6 | 2,6 | 2,6 | 2,7 |
| England/Wales | 41,0 | 40,8 | 41,2 | 42,4 | 42,2 | 42,6 | 42,7 | 42,5 | 42,9 | 1,7 | 1,4 | 1,9 |
| Netherlands | 41,3 | 41,2 | 41,4 | 42,2 | 42,1 | 42,3 | 42,7 | 42,6 | 42,9 | 1,4 | 1,3 | 1,6 |
| Belgium | 41,2 | 41,1 | 41,2 | 41,9 | 41,9 | 42,0 | 42,6 | 42,5 | 42,6 | 1,4 | 1,3 | 1,5 |
| Austria | 41,5 | 41,4 | 41,5 | 42,2 | 42,2 | 42,3 | 42,8 | 42,7 | 42,9 | 1,3 | 1,2 | 1,5 |
| Switzerland | 41,9 | 41,9 | 42,0 | 42,9 | 42,8 | 42,9 | 43,1 | 43,0 | 43,2 | 1,2 | 1,1 | 1,3 |
| France | 41,5 | 41,3 | 41,7 | 42,3 | 42,1 | 42,5 | 43,1 | 42,8 | 43,3 | 1,6 | 1,3 | 1,9 |
| Spain | 42,4 | 42,4 | 42,4 | 42,7 | 42,7 | 42,8 | 42,9 | 42,9 | 43,0 | 0,6 | 0,5 | 0,6 |
| Hungary | 39,0 | 39,0 | 39,1 | 41,3 | 41,2 | 41,3 | 41,9 | 41,8 | 42,0 | 2,9 | 2,8 | 2,9 |
| Poland | 39,8 | 39,8 | 39,8 | 41,5 | 41,5 | 41,5 | 42,5 | 42,5 | 42,5 | 2,7 | 2,7 | 2,7 |
| Lithuania | 37,8 | 37,6 | 38,1 | 40,9 | 40,9 | 41,0 | 42,3 | 42,2 | 42,4 | 4,5 | 4,2 | 4,7 |
| Estonia | 38,7 | 38,4 | 39,0 | 41,4 | 41,3 | 41,5 | 42,6 | 42,5 | 42,7 | 3,9 | 3,6 | 4,2 |
| **Average** | 41,0 | 40,9 | 41,1 | 42,1 | 42,0 | 42,2 | 42,7 | 42,5 | 42,8 | 1,7 | 1,5 | 1,8 |

**Appendix table F1. Educational inequalities in risk factor prevalence**

| **A. Men** |  |  |  |  |  |
| --- | --- | --- | --- | --- | --- |
| **Country** | **Risk Factor** | **Prevalence Ratio*** | **95% CI** | **Age Standardized Prevalence**** | **95% CI** |
| Finland | Father's manual occupation | 1.4 | [1.1,1.8] | 56.9 | [53.0,60.9] |
| Finland | Low income | 4.8 | [3.4,6.2] | 14.4 | [13.7,15.2] |
| Finland | Few social contacts | 1.6 | [1.1,2.2] | 39.9 | [36.3,43.4] |
| Finland | Smoking | 2.9 | [1.6,4.2] | 28.3 | [24.9,31.6] |
| Finland | High alcohol consumption | 0.8 | [0.1,1.5] | 10.3 | [8.1,12.6] |
| Finland | High body-weight | 1.1 | [0.9,1.3] | 65.3 | [61.7,68.8] |
| Finland | Low physical activity | 1.1 | [0.9,1.4] | 66.1 | [62.7,69.6] |
| Finland | Low fruits and vegetables | 1.4 | [1.0,1.7] | 47.2 | [43.5,50.9] |
| Sweden | Father's manual occupation | 1.8 | [1.2,2.3] | 47.1 | [42.9,51.4] |
| Sweden | Low income | 3.1 | [2.1,4.2] | 12.3 | [11.3,13.3] |
| Sweden | Few social contacts | 1.2 | [0.7,1.7] | 29.3 | [25.7,32.8] |
| Sweden | Smoking | 5.3 | [1.0,9.6] | 15.3 | [12.3,18.2] |
| Sweden | High alcohol consumption | 1.3 | [0.5,2.2] | 14.0 | [11.2,16.7] |
| Sweden | High body-weight | 1.5 | [1.1,1.8] | 66.1 | [62.3,69.8] |
| Sweden | Low physical activity | 1.1 | [0.9,1.3] | 68.3 | [64.6,72.0] |
| Sweden | Low fruits and vegetables | 1.6 | [1.2,2.0] | 58.9 | [55.0,62.7] |
| Norway | Father's manual occupation | 1.8 | [1.3,2.2] | 45.9 | [41.4,50.5] |
| Norway | Low income | 4.4 | [3.1,5.7] | 11.4 | [10.5,12.2] |
| Norway | Few social contacts | 1.0 | [0.6,1.5] | 35.0 | [30.8,39.2] |
| Norway | Smoking | 3.3 | [1.5,5.2] | 23.0 | [19.3,26.8] |
| Norway | High alcohol consumption | 0.5 | [0.0,0.9] | 13.2 | [10.2,16.2] |
| Norway | High body-weight | 1.0 | [0.8,1.3] | 68.4 | [64.3,72.5] |
| Norway | Low physical activity | 1.1 | [0.9,1.2] | 71.9 | [68.0,75.9] |
| Norway | Low fruits and vegetables | 1.8 | [1.2,2.3] | 44.5 | [40.1,48.9] |
| Denmark | Father's manual occupation | 2.1 | [1.5,2.7] | 51.0 | [46.3,55.7] |
| Denmark | Low income | 3.4 | [1.5,5.2] | 13.3 | [12.3,14.3] |
| Denmark | Few social contacts | 1.1 | [0.7,1.5] | 33.4 | [29.1,37.6] |
| Denmark | Smoking | 2.2 | [1.3,3.1] | 26.4 | [22.3,30.4] |
| Denmark | High alcohol consumption | 1.5 | [0.8,2.3] | 21.7 | [17.9,25.5] |
| Denmark | High body-weight | 1.5 | [1.1,1.8] | 59.5 | [55.0,63.9] |
| Denmark | Low physical activity | 0.9 | [0.7,1.1] | 65.5 | [61.2,69.8] |
| Denmark | Low fruits and vegetables | 2.0 | [1.5,2.5] | 55.0 | [50.5,59.4] |
| England/Wales | Father's manual occupation | 1.6 | [1.3,1.9] | 57.0 | [53.3,60.7] |
| England/Wales | Low income | 2.8 | [2.3,3.2] | 17.4 | [16.5,18.3] |
| England/Wales | Few social contacts | 1.0 | [0.8,1.2] | 47.7 | [44.2,51.2] |
| England/Wales | Smoking | 2.1 | [1.2,3.0] | 22.5 | [19.6,25.5] |
| England/Wales | High alcohol consumption | 1.1 | [0.7,1.5] | 30.8 | [27.6,34.1] |
| England/Wales | High body-weight | 1.3 | [1.1,1.5] | 66.3 | [62.9,69.6] |
| England/Wales | Low physical activity | 1.2 | [1.0,1.4] | 60.8 | [57.3,64.2] |
| England/Wales | Low fruits and vegetables | 1.9 | [1.3,2.4] | 40.7 | [37.3,44.2] |
| Netherlands | Father's manual occupation | 1.7 | [1.2,2.1] | 46.3 | [42.3,50.4] |
| Netherlands | Low income | 2.7 | [1.6,3.8] | 16.1 | [15.3,17.0] |
| Netherlands | Few social contacts | 1.1 | [0.7,1.5] | 31.9 | [28.4,35.4] |
| Netherlands | Smoking | 2.3 | [1.3,3.3] | 31.2 | [27.7,34.8] |
| Netherlands | High alcohol consumption | 1.0 | [0.5,1.5] | 17.5 | [14.6,20.5] |
| Netherlands | High body-weight | 1.3 | [1.1,1.6] | 59.9 | [56.1,63.6] |
| Netherlands | Low physical activity | 0.9 | [0.8,1.1] | 66.2 | [62.6,69.8] |
| Netherlands | Low fruits and vegetables | 1.2 | [0.9,1.5] | 45.8 | [41.9,49.6] |
| Belgium | Father's manual occupation | 2.2 | [1.6,2.8] | 53.4 | [49.0,57.9] |
| Belgium | Low income | 3.4 | [2.6,4.3] | 16.7 | [15.6,17.8] |
| Belgium | Few social contacts | 1.0 | [0.7,1.3] | 36.6 | [32.7,40.5] |
| Belgium | Smoking | 3.3 | [1.7,4.9] | 29.1 | [25.3,32.9] |
| Belgium | High alcohol consumption | 0.6 | [0.4,0.9] | 20.3 | [17.0,23.6] |
| Belgium | High body-weight | 1.1 | [0.9,1.3] | 58.4 | [54.4,62.5] |
| Belgium | Low physical activity | 0.9 | [0.8,1.0] | 68.6 | [64.8,72.4] |
| Belgium | Low fruits and vegetables | 1.6 | [1.1,2.0] | 44.6 | [40.5,48.6] |
| Austria | Father's manual occupation | 2.1 | [1.4,2.8] | 51.9 | [47.7,56.0] |
| Austria | Low income | 2.6 | [2.0,3.2] | 17.1 | [15.9,18.3] |
| Austria | Few social contacts | 1.2 | [0.8,1.7] | 39.8 | [36.0,43.6] |
| Austria | Smoking | 1.8 | [0.9,2.7] | 33.8 | [30.2,37.5] |
| Austria | High alcohol consumption | 1.1 | [0.5,1.7] | 23.1 | [19.8,26.4] |
| Austria | High body-weight | 1.5 | [1.2,1.9] | 65.5 | [61.8,69.2] |
| Austria | Low physical activity | 1.6 | [1.2,1.9] | 66.5 | [62.8,70.2] |
| Austria | Low fruits and vegetables | 1.0 | [0.8,1.2] | 62.3 | [58.6,66.0] |
| Switzerland | Father's manual occupation | 1.7 | [1.2,2.2] | 44.4 | [39.9,48.9] |
| Switzerland | Low income | 4.5 | [3.4,5.6] | 15.9 | [15.0,16.9] |
| Switzerland | Few social contacts | 1.4 | [0.9,1.8] | 38.2 | [34.1,42.3] |
| Switzerland | Smoking | 2.3 | [1.2,3.4] | 27.9 | [24.1,31.8] |
| Switzerland | High alcohol consumption | 0.6 | [0.2,1.0] | 16.9 | [13.7,20.1] |
| Switzerland | High body-weight | 1.2 | [0.9,1.5] | 57.8 | [53.5,62.0] |
| Switzerland | Low physical activity | 0.9 | [0.7,1.0] | 63.1 | [59.0,67.3] |
| Switzerland | Low fruits and vegetables | 1.4 | [0.8,1.9] | 44.0 | [39.8,48.3] |
| France | Father's manual occupation | 2.3 | [1.5,3.2] | 53.1 | [49.0,57.3] |
| France | Low income | 4.7 | [3.6,5.8] | 16.9 | [16.0,17.8] |
| France | Few social contacts | 1.3 | [0.8,1.7] | 42.2 | [38.4,46.0] |
| France | Smoking | 2.1 | [1.1,3.1] | 31.4 | [27.9,34.9] |
| France | High alcohol consumption | 1.9 | [0.8,3.0] | 19.6 | [16.5,22.6] |
| France | High body-weight | 1.4 | [1.0,1.7] | 60.7 | [56.9,64.4] |
| France | Low physical activity | 1.0 | [0.8,1.1] | 74.5 | [71.2,77.8] |
| France | Low fruits and vegetables | 1.3 | [0.9,1.6] | 46.5 | [42.9,50.2] |
| Spain | Father's manual occupation | 1.8 | [1.3,2.3] | 50.0 | [46.0,54.1] |
| Spain | Low income | 3.7 | [2.9,4.4] | 19.2 | [18.5,20.0] |
| Spain | Few social contacts | 1.1 | [0.8,1.5] | 27.0 | [23.6,30.4] |
| Spain | Smoking | 1.8 | [1.2,2.4] | 33.3 | [29.7,36.8] |
| Spain | High alcohol consumption | 1.7 | [0.8,2.5] | 19.5 | [16.5,22.6] |
| Spain | High body-weight | 1.2 | [1.1,1.4] | 70.1 | [66.6,73.6] |
| Spain | Low physical activity | 1.0 | [0.8,1.1] | 61.5 | [57.8,65.3] |
| Spain | Low fruits and vegetables | 1.2 | [1.0,1.4] | 57.0 | [53.2,60.8] |
| Hungary | Father's manual occupation | 1.3 | [1.0,1.7] | 65.9 | [61.5,70.3] |
| Hungary | Low income | 16.0 | [8.9,23.1] | 19.0 | [18.0,19.9] |
| Hungary | Few social contacts | 1.0 | [0.8,1.2] | 72.0 | [68.2,75.8] |
| Hungary | Smoking | 3.1 | [1.6,4.7] | 44.9 | [40.5,49.2] |
| Hungary | High alcohol consumption | 1.1 | [0.4,1.8] | 21.1 | [17.5,24.8] |
| Hungary | High body-weight | 0.8 | [0.6,1.0] | 71.9 | [68.0,75.8] |
| Hungary | Low physical activity | 1.1 | [0.9,1.2] | 81.2 | [77.8,84.6] |
| Hungary | Low fruits and vegetables | 1.3 | [1.0,1.5] | 76.3 | [72.6,80.0] |
| Poland | Father's manual occupation | 1.2 | [0.9,1.6] | 50.0 | [45.5,54.6] |
| Poland | Low income | 10.3 | [7.1,13.5] | 19.6 | [18.9,20.4] |
| Poland | Few social contacts | 0.8 | [0.7,1.0] | 66.2 | [62.1,70.2] |
| Poland | Smoking | 2.6 | [1.3,3.9] | 36.5 | [32.4,40.5] |
| Poland | High alcohol consumption | 1.6 | [0.4,2.8] | 13.6 | [10.7,16.5] |
| Poland | High body-weight | 1.0 | [0.8,1.2] | 70.5 | [66.6,74.4] |
| Poland | Low physical activity | 0.9 | [0.7,1.1] | 61.4 | [57.2,65.5] |
| Poland | Low fruits and vegetables | 1.1 | [0.8,1.4] | 46.9 | [42.6,51.1] |
| Lithuania | Father's manual occupation | 1.2 | [0.9,1.6] | 59.9 | [55.5,64.3] |
| Lithuania | Low income | 9.0 | [4.5,13.5] | 17.7 | [16.4,19.0] |
| Lithuania | Few social contacts | 1.0 | [0.8,1.2] | 70.5 | [67.0,74.0] |
| Lithuania | Smoking | 1.5 | [1.0,2.0] | 49.9 | [46.0,53.8] |
| Lithuania | High alcohol consumption | 2.7 | [1.3,4.1] | 21.9 | [18.6,25.2] |
| Lithuania | High body-weight | 1.0 | [0.7,1.2] | 68.3 | [64.6,71.9] |
| Lithuania | Low physical activity | 0.8 | [0.6,1.0] | 53.8 | [49.9,57.7] |
| Lithuania | Low fruits and vegetables | 1.9 | [1.4,2.4] | 61.5 | [57.7,65.2] |
| Estonia | Father's manual occupation | 1.3 | [1.0,1.7] | 59.8 | [55.6,64.1] |
| Estonia | Low income | 2.8 | [2.1,3.6] | 18.5 | [17.3,19.7] |
| Estonia | Few social contacts | 0.8 | [0.6,1.0] | 63.0 | [59.3,66.8] |
| Estonia | Smoking | 3.4 | [1.9,4.9] | 39.9 | [36.1,43.7] |
| Estonia | High alcohol consumption | 0.7 | [0.0,1.4] | 12.7 | [10.1,15.4] |
| Estonia | High body-weight | 0.9 | [0.7,1.1] | 63.2 | [59.4,67.0] |
| Estonia | Low physical activity | 0.8 | [0.6,1.0] | 54.3 | [50.4,58.2] |
| Estonia | Low fruits and vegetables | 1.3 | [1.0,1.7] | 52.3 | [48.4,56.1] |
| Average | Father's manual occupation | 1.7 | [1.5,1.8] | 53.0 | [51.9,54.2] |
| Average | Low income | 4.0 | [3.7,4.4] | 16.8 | [16.6,17.1] |
| Average | Few social contacts | 1.0 | [0.9,1.1] | 43.8 | [42.8,44.9] |
| Average | Smoking | 2.3 | [1.9,2.7] | 30.3 | [29.4,31.3] |
| Average | High alcohol consumption | 1.1 | [0.9,1.4] | 20.8 | [19.9,21.6] |
| Average | High body-weight | 1.2 | [1.1,1.3] | 65.4 | [64.4,66.4] |
| Average | Low physical activity | 1.0 | [1.0,1.1] | 65.7 | [64.7,66.7] |
| Average | Low fruits and vegetables | 1.4 | [1.3,1.6] | 49.0 | [47.9,50.0] |

*Prevalence ratio was estimated using logistic regression and the STATA post-estimation command adjrr. For the three level risk factors, smoking is an estimate of never smokers vs current and former smokers; high body-weight compares individuals with normal weight vs overweight and obese, and for high alcohol consumption, it compares the <25 g/day to the other two risk categories.

**Age-standardized prevalence was obtained using direct standardization with the 2013 European Standard Population

| **B. Women** |  |  |  |  |  |
| --- | --- | --- | --- | --- | --- |
| **Country** | **Risk Factor** | **Prevalence Ratio*** | **95% CI** | **Prevalence**** | **95% CI** |
| Finland | Father's manual occupation | 1.5 | [1.1,1.9] | 56.4 | [52.4,60.4] |
| Finland | Low income | 3.9 | [2.8,4.9] | 15.4 | [14.6,16.2] |
| Finland | Few social contacts | 1.1 | [0.7,1.5] | 35.6 | [32.1,39.2] |
| Finland | Smoking | 4.6 | [2.1,7.2] | 21.8 | [18.7,24.9] |
| Finland | High alcohol consumption | 0.7 | [-0.1,1.4] | 2.7 | [1.5,3.8] |
| Finland | High body-weight | 1.6 | [1.2,2.1] | 51.7 | [47.9,55.5] |
| Finland | Low physical activity | 0.9 | [0.7,1.1] | 58.5 | [54.9,62.2] |
| Finland | Low fruits and vegetables | 2.5 | [1.5,3.6] | 31.6 | [28.2,35.0] |
| Sweden | Father's manual occupation | 1.9 | [1.4,2.4] | 54.5 | [50.2,58.9] |
| Sweden | Low income | 3.0 | [2.2,3.9] | 15.7 | [14.6,16.8] |
| Sweden | Few social contacts | 1.1 | [0.6,1.6] | 26.0 | [22.4,29.6] |
| Sweden | Smoking | 2.6 | [0.7,4.4] | 15.3 | [12.3,18.3] |
| Sweden | High alcohol consumption | 0.4 | [-0.1,0.9] | 3.9 | [2.3,5.4] |
| Sweden | High body-weight | 1.6 | [1.2,2.1] | 49.5 | [45.3,53.6] |
| Sweden | Low physical activity | 1.1 | [0.9,1.3] | 68.2 | [64.4,72.0] |
| Sweden | Low fruits and vegetables | 2.3 | [1.4,3.1] | 36.4 | [32.4,40.3] |
| Norway | Father's manual occupation | 2.0 | [1.5,2.5] | 48.9 | [44.3,53.5] |
| Norway | Low income | 4.6 | [3.4,5.7] | 15.4 | [14.4,16.3] |
| Norway | Few social contacts | 1.8 | [1.1,2.5] | 36.6 | [32.4,40.8] |
| Norway | Smoking | 5.4 | [1.9,8.9] | 22.9 | [19.2,26.7] |
| Norway | High alcohol consumption | 0.8 | [-0.3,1.9] | 3.7 | [2.0,5.3] |
| Norway | High body-weight | 1.6 | [1.1,2.1] | 45.5 | [41.0,50.0] |
| Norway | Low physical activity | 1.1 | [0.9,1.3] | 74.1 | [70.3,77.9] |
| Norway | Low fruits and vegetables | 1.8 | [1.0,2.6] | 29.6 | [25.5,33.7] |
| Denmark | Father's manual occupation | 1.7 | [1.3,2.1] | 52.1 | [47.7,56.4] |
| Denmark | Low income | 3.4 | [1.7,5.1] | 12.7 | [11.7,13.7] |
| Denmark | Few social contacts | 1.5 | [1.0,2.0] | 34.5 | [30.5,38.5] |
| Denmark | Smoking | 2.7 | [1.5,4.0] | 22.6 | [19.2,26.0] |
| Denmark | High alcohol consumption | 1.0 | [0.2,1.8] | 7.9 | [5.5,10.2] |
| Denmark | High body-weight | 1.7 | [1.2,2.2] | 43.1 | [38.9,47.3] |
| Denmark | Low physical activity | 1.1 | [0.9,1.3] | 66.0 | [62.0,70.0] |
| Denmark | Low fruits and vegetables | 2.7 | [1.6,3.7] | 30.0 | [26.2,33.9] |
| England/Wales | Father's manual occupation | 2.3 | [1.7,2.8] | 50.4 | [46.8,54.1] |
| England/Wales | Low income | 3.0 | [2.6,3.5] | 18.9 | [17.9,19.8] |
| England/Wales | Few social contacts | 0.9 | [0.7,1.2] | 40.6 | [37.3,43.8] |
| England/Wales | Smoking | 2.6 | [1.4,3.7] | 20.3 | [17.6,23.0] |
| England/Wales | High alcohol consumption | 0.7 | [0.4,1.0] | 15.0 | [12.6,17.4] |
| England/Wales | High body-weight | 1.3 | [1.0,1.5] | 52.3 | [48.8,55.7] |
| England/Wales | Low physical activity | 1.0 | [0.9,1.2] | 65.7 | [62.5,68.9] |
| England/Wales | Low fruits and vegetables | 2.4 | [1.7,3.2] | 32.3 | [29.2,35.4] |
| Netherlands | Father's manual occupation | 2.1 | [1.6,2.6] | 50.4 | [46.5,54.4] |
| Netherlands | Low income | 3.1 | [2.2,4.1] | 18.1 | [17.2,19.0] |
| Netherlands | Few social contacts | 1.3 | [0.9,1.7] | 28.0 | [24.7,31.3] |
| Netherlands | Smoking | 3.1 | [1.7,4.5] | 22.3 | [19.3,25.4] |
| Netherlands | High alcohol consumption | 1.8 | [0.2,3.5] | 5.4 | [3.7,7.1] |
| Netherlands | High body-weight | 1.7 | [1.3,2.1] | 48.8 | [45.1,52.5] |
| Netherlands | Low physical activity | 1.0 | [0.8,1.1] | 63.9 | [60.3,67.4] |
| Netherlands | Low fruits and vegetables | 1.7 | [1.2,2.2] | 36.0 | [32.5,39.5] |
| Belgium | Father's manual occupation | 2.1 | [1.6,2.7] | 54.6 | [50.4,58.8] |
| Belgium | Low income | 4.3 | [3.4,5.2] | 19.0 | [17.8,20.2] |
| Belgium | Few social contacts | 1.3 | [0.9,1.7] | 36.5 | [32.7,40.2] |
| Belgium | Smoking | 3.3 | [1.8,4.7] | 26.6 | [23.1,30.1] |
| Belgium | High alcohol consumption | 0.9 | [0.2,1.6] | 6.9 | [5.0,8.8] |
| Belgium | High body-weight | 1.3 | [0.9,1.6] | 44.0 | [40.1,47.8] |
| Belgium | Low physical activity | 0.9 | [0.8,1.0] | 75.0 | [71.5,78.4] |
| Belgium | Low fruits and vegetables | 1.4 | [0.9,1.8] | 35.3 | [31.5,39.0] |
| Austria | Father's manual occupation | 3.0 | [1.7,4.2] | 52.9 | [48.9,57.0] |
| Austria | Low income | 3.5 | [2.7,4.3] | 20.6 | [19.3,21.9] |
| Austria | Few social contacts | 1.5 | [1.0,2.0] | 43.8 | [40.0,47.5] |
| Austria | Smoking | 2.6 | [1.1,4.0] | 29.4 | [25.9,32.9] |
| Austria | High alcohol consumption | 0.8 | [-0.1,1.7] | 4.3 | [2.7,5.8] |
| Austria | High body-weight | 1.9 | [1.1,2.6] | 42.1 | [38.4,45.8] |
| Austria | Low physical activity | 1.3 | [1.0,1.5] | 68.6 | [65.1,72.1] |
| Austria | Low fruits and vegetables | 1.6 | [1.1,2.2] | 47.2 | [43.4,51.0] |
| Switzerland | Father's manual occupation | 1.8 | [1.2,2.4] | 45.8 | [41.5,50.1] |
| Switzerland | Low income | 3.0 | [2.4,3.6] | 19.7 | [18.7,20.7] |
| Switzerland | Few social contacts | 1.5 | [0.7,2.2] | 30.6 | [26.8,34.3] |
| Switzerland | Smoking | 2.1 | [1.0,3.1] | 25.0 | [21.5,28.6] |
| Switzerland | High alcohol consumption | 0.4 | [0.0,0.8] | 4.5 | [2.8,6.1] |
| Switzerland | High body-weight | 2.2 | [1.1,3.2] | 33.8 | [29.9,37.7] |
| Switzerland | Low physical activity | 1.1 | [0.8,1.4] | 56.8 | [52.8,60.9] |
| Switzerland | Low fruits and vegetables | 0.9 | [0.4,1.4] | 21.6 | [18.2,25.0] |
| France | Father's manual occupation | 1.8 | [1.2,2.4] | 51.7 | [47.7,55.8] |
| France | Low income | 6.3 | [5.0,7.5] | 18.7 | [17.8,19.6] |
| France | Few social contacts | 2.2 | [1.2,3.2] | 38.1 | [34.5,41.6] |
| France | Smoking | 1.9 | [0.9,2.9] | 28.0 | [24.8,31.1] |
| France | High alcohol consumption | 0.9 | [-0.2,2.1] | 3.6 | [2.2,4.9] |
| France | High body-weight | 2.4 | [1.4,3.4] | 44.8 | [41.1,48.5] |
| France | Low physical activity | 0.9 | [0.8,1.1] | 80.8 | [78.0,83.6] |
| France | Low fruits and vegetables | 1.6 | [0.9,2.3] | 32.8 | [29.4,36.2] |
| Spain | Father's manual occupation | 1.4 | [1.1,1.8] | 49.0 | [45.0,53.1] |
| Spain | Low income | 3.7 | [3.0,4.3] | 19.7 | [19.0,20.5] |
| Spain | Few social contacts | 1.4 | [1.0,1.8] | 36.2 | [32.5,39.9] |
| Spain | Smoking | 1.9 | [1.2,2.6] | 29.0 | [25.5,32.4] |
| Spain | High alcohol consumption | 0.6 | [-0.1,1.2] | 2.6 | [1.4,3.8] |
| Spain | High body-weight | 1.5 | [1.1,1.9] | 46.6 | [42.8,50.4] |
| Spain | Low physical activity | 1.1 | [0.9,1.3] | 64.0 | [60.4,67.6] |
| Spain | Low fruits and vegetables | 1.4 | [1.0,1.8] | 42.0 | [38.3,45.7] |
| Hungary | Father's manual occupation | 1.5 | [1.2,1.8] | 67.6 | [63.7,71.6] |
| Hungary | Low income | 13.6 | [8.8,18.3] | 19.2 | [18.2,20.1] |
| Hungary | Few social contacts | 0.9 | [0.8,1.1] | 77.7 | [74.4,81.1] |
| Hungary | Smoking | 2.8 | [1.3,4.2] | 28.7 | [25.0,32.3] |
| Hungary | High alcohol consumption | 0.1 | [-0.1,0.3] | 1.3 | [0.4,2.3] |
| Hungary | High body-weight | 1.3 | [1.0,1.6] | 58.4 | [54.4,62.4] |
| Hungary | Low physical activity | 1.0 | [0.9,1.2] | 82.3 | [79.2,85.4] |
| Hungary | Low fruits and vegetables | 1.5 | [1.2,1.7] | 71.8 | [68.2,75.4] |
| Poland | Father's manual occupation | 1.0 | [0.8,1.2] | 56.1 | [51.9,60.3] |
| Poland | Low income | 10.8 | [8.2,13.3] | 19.4 | [18.7,20.1] |
| Poland | Few social contacts | 1.0 | [0.8,1.2] | 65.6 | [61.8,69.4] |
| Poland | Smoking | 2.0 | [1.0,2.9] | 24.3 | [20.8,27.7] |
| Poland | High alcohol consumption | 0.7 | [-0.4,1.8] | 1.3 | [0.4,2.2] |
| Poland | High body-weight | 1.1 | [0.8,1.4] | 49.8 | [45.9,53.7] |
| Poland | Low physical activity | 1.2 | [1.0,1.4] | 64.5 | [60.7,68.3] |
| Poland | Low fruits and vegetables | 2.3 | [1.4,3.3] | 33.0 | [29.2,36.7] |
| Lithuania | Father's manual occupation | 1.1 | [0.8,1.4] | 56.4 | [52.4,60.4] |
| Lithuania | Low income | 6.3 | [4.2,8.3] | 19.5 | [18.3,20.8] |
| Lithuania | Few social contacts | 1.1 | [0.9,1.3] | 71.7 | [68.6,74.8] |
| Lithuania | Smoking | 3.0 | [1.1,4.9] | 17.6 | [15.0,20.3] |
| Lithuania | High alcohol consumption | 2.0 | [-0.5,4.4] | 3.3 | [2.0,4.7] |
| Lithuania | High body-weight | 1.3 | [1.0,1.5] | 57.4 | [54.0,60.7] |
| Lithuania | Low physical activity | 0.9 | [0.7,1.0] | 63.0 | [59.8,66.3] |
| Lithuania | Low fruits and vegetables | 2.5 | [1.8,3.1] | 47.2 | [43.9,50.6] |
| Estonia | Father's manual occupation | 0.9 | [0.7,1.1] | 59.9 | [56.0,63.9] |
| Estonia | Low income | 2.4 | [1.9,2.9] | 19.5 | [18.4,20.6] |
| Estonia | Few social contacts | 1.2 | [0.9,1.4] | 62.5 | [59.0,65.9] |
| Estonia | Smoking | 3.6 | [1.9,5.3] | 22.1 | [19.1,25.1] |
| Estonia | High alcohol consumption | 3.4 | [-2.9,9.6] | 1.7 | [0.8,2.7] |
| Estonia | High body-weight | 1.3 | [1.1,1.6] | 52.4 | [49.0,55.7] |
| Estonia | Low physical activity | 1.1 | [0.9,1.3] | 54.7 | [51.1,58.2] |
| Estonia | Low fruits and vegetables | 1.8 | [1.3,2.2] | 35.5 | [32.2,38.9] |
| Average | Father's manual occupation | 1.6 | [1.5,1.8] | 52.5 | [51.4,53.6] |
| Average | Low income | 4.1 | [3.9,4.4] | 18.2 | [18.0,18.5] |
| Average | Few social contacts | 1.2 | [1.0,1.3] | 43.0 | [42.0,44.0] |
| Average | Smoking | 2.4 | [2.0,2.9] | 24.9 | [24.0,25.8] |
| Average | High alcohol consumption | 0.6 | [0.4,0.7] | 5.9 | [5.4,6.4] |
| Average | High body-weight | 1.4 | [1.3,1.6] | 47.9 | [46.9,48.9] |
| Average | Low physical activity | 1.1 | [1.0,1.1] | 69.1 | [68.2,70.1] |
| Average | Low fruits and vegetables | 1.9 | [1.7,2.2] | 35.8 | [34.8,36.8] |

risk factors, smoking is an estimate of never smokers vs current and former smokers; high body-weight compares individuals with normal weight vs overweight and obese, and for high alcohol consumption, it compares the <25 g/day to the other two risk categories.

**Age-standardized prevalence was obtained using direct standardization with the 2013 European Standard Population

**Appendix table G1. Effect of ‘upward levelling’ of risk factor prevalence on partial disability-free life expectancy of low educated**

|  |  |  |  |  |  |  |  |  | |
| --- | --- | --- | --- | --- | --- | --- | --- | --- | --- |
| Country | Father's Manual Occupation | Low income | Few social contacts | Smoking | High alcohol consumption | High body-weight | Low physical activity | | Low fruits & vegetables |
| Men |  |  |  |  |  |  |  | |  |
| Finland | 0.7 | 1.6 | 0.2 | 1.4 | -0.1 | 0.9 | 0.4 | | 0.4 |
|  | [0.6,0.8] | [1.5,1.7] | [0.1,0.3] | [1.3,1.5] | [-0.3,0.1] | [0.8,1.0] | [0.3,0.5] | | [0.3,0.5] |
| Sweden | 0.7 | 0.7 | 0.0 | 1.1 | 0.0 | 1.3 | 0.3 | | 0.6 |
|  | [0.6,0.8] | [0.6,0.8] | [-0.1,0.1] | [1.0,1.2] | [-0.1,0.2] | [1.2,1.4] | [0.2,0.4] | | [0.5,0.7] |
| Norway | 0.8 | 0.9 | 0.0 | 1.5 | -0.1 | 0.5 | 0.2 | | 0.7 |
|  | [0.7,0.9] | [0.8,1.0] | [-0.1,0.1] | [1.3,1.6] | [-0.3,0.1] | [0.4,0.6] | [0.0,0.3] | | [0.5,0.8] |
| Denmark | 1.1 | 1.0 | 0.0 | 1.2 | 0.1 | 1.5 | -0.4 | | 1.0 |
|  | [1.0,1.2] | [0.9,1.1] | [-0.1,0.1] | [1.1,1.3] | [-0.1,0.4] | [1.4,1.6] | [-0.5,-0.3] | | [0.9,1.1] |
| Eng/Wales | 0.9 | 1.2 | 0.0 | 0.8 | 0.0 | 1.0 | 0.5 | | 0.7 |
|  | [0.8,1.0] | [1.0,1.3] | [-0.1,0.1] | [0.7,1.0] | [-0.2,0.2] | [0.9,1.1] | [0.4,0.6] | | [0.6,0.8] |
| Netherlands | 0.8 | 1.2 | 0.0 | 1.0 | 0.0 | 1.1 | -0.2 | | 0.2 |
|  | [0.7,0.9] | [1.1,1.3] | [-0.1,0.1] | [0.9,1.2] | [-0.1,0.1] | [1.0,1.2] | [-0.3,-0.1] | | [0.1,0.3] |
| Belgium | 1.3 | 1.5 | 0.0 | 1.3 | -0.1 | 0.5 | -0.3 | | 0.5 |
|  | [1.2,1.4] | [1.4,1.6] | [-0.1,0.1] | [1.2,1.5] | [-0.3,0.1] | [0.4,0.6] | [-0.4,-0.2] | | [0.4,0.6] |
| Austria | 1.6 | 1.8 | 0.1 | 1.0 | 0.0 | 2.8 | 1.5 | | -0.1 |
|  | [1.5,1.7] | [1.7,1.9] | [0.0,0.2] | [0.9,1.1] | [-0.2,0.3] | [2.7,2.9] | [1.4,1.6] | | [-0.2,-0.0] |
| Switzerland | 0.8 | 1.7 | 0.1 | 1.1 | -0.1 | 1.1 | -0.4 | | 0.3 |
|  | [0.7,0.9] | [1.6,1.8] | [0.0,0.2] | [0.9,1.2] | [-0.3,0.1] | [1.0,1.2] | [-0.5,-0.3] | | [0.2,0.4] |
| France | 1.1 | 1.2 | 0.1 | 1.0 | 0.1 | 1.1 | 0.0 | | 0.3 |
|  | [1.0,1.3] | [1.1,1.3] | [0.0,0.2] | [0.8,1.1] | [-0.1,0.3] | [1.0,1.2] | [-0.1,0.1] | | [0.2,0.4] |
| Spain | 0.8 | 1.0 | 0.0 | 0.5 | 0.1 | 0.9 | -0.1 | | 0.3 |
|  | [0.7,0.9] | [0.9,1.1] | [-0.1,0.1] | [0.4,0.6] | [-0.1,0.2] | [0.8,1.0] | [-0.2,-0.0] | | [0.2,0.4] |
| Hungary | 0.7 | 2.3 | 0.0 | 2.4 | 0.0 | -0.7 | 0.2 | | 0.5 |
|  | [0.6,0.8] | [2.2,2.5] | [-0.1,0.1] | [2.3,2.5] | [-0.3,0.3] | [-0.8,-0.6] | [0.1,0.3] | | [0.4,0.6] |
| Poland | 0.3 | 2.2 | -0.1 | 0.9 | 0.1 | 0.5 | -0.3 | | 0.1 |
|  | [0.2,0.4] | [2.1,2.3] | [-0.2,-0.0] | [0.8,1.0] | [-0.1,0.2] | [0.4,0.6] | [-0.4,-0.2] | | [0.0,0.2] |
| Lithuania | 0.5 | 2.2 | 0.1 | 1.3 | 0.5 | 0.6 | -0.7 | | 1.2 |
|  | [0.4,0.6] | [2.1,2.3] | [0.0,0.2] | [1.2,1.4] | [0.1,0.8] | [0.5,0.7] | [-0.8,-0.6] | | [1.1,1.3] |
| Estonia | 0.8 | 1.8 | -0.1 | 2.3 | 0.0 | -0.1 | -0.8 | | 0.6 |
|  | [0.6,0.9] | [1.7,1.9] | [-0.2,-0.0] | [2.2,2.4] | [-0.2,0.2] | [-0.2,-0.0] | [-0.9,-0.7] | | [0.5,0.7] |
| Average | 0.9 | 1.4 | 0.0 | 0.9 | 0.0 | 0.9 | 0.1 | | 0.4 |
|  | [0.7,0.9] | [1.3,1.5] | [-0.1,0.1] | [0.8,1.1] | [-0.1,0.2] | [0.8,1.0] | [0.0,0.2] | | [0.3,0.5] |
| Women |  |  |  |  |  |  |  | |  |
| Finland | 0.9 | 1.6 | 0.0 | 1.4 | 0.0 | 1.8 | -0.2 | | 0.9 |
|  | [0.8,1.0] | [1.5,1.7] | [-0.1,0.1] | [1.3,1.6] | [-0.1,0.1] | [1.7,1.9] | [-0.3,-0.1] | | [0.8,1.0] |
| Sweden | 1.2 | 1.1 | 0.0 | -0.1 | 0.0 | 1.6 | 0.2 | | 0.9 |
|  | [1.1,1.3] | [1.0,1.2] | [-0.1,0.1] | [-0.2,-0.0] | [-0.2,0.1] | [1.5,1.7] | [0.1,0.3] | | [0.8,1.0] |
| Norway | 1.3 | 1.6 | 0.2 | 1.6 | 0.0 | 1.6 | 0.3 | | 0.6 |
|  | [1.2,1.4] | [1.5,1.7] | [0.1,0.3] | [1.5,1.7] | [-0.2,0.1] | [1.5,1.7] | [0.2,0.4] | | [0.5,0.7] |
| Denmark | 0.9 | 1.1 | 0.1 | 1.1 | 0.0 | 1.5 | 0.3 | | 0.9 |
|  | [0.8,1.0] | [1.0,1.2] | [0.0,0.2] | [1.0,1.2] | [-0.1,0.1] | [1.4,1.6] | [0.2,0.4] | | [0.8,1.0] |
| Eng/Wales | 1.4 | 1.4 | 0.0 | 0.9 | 0.0 | 0.9 | 0.1 | | 0.7 |
|  | [1.3,1.6] | [1.3,1.5] | [-0.1,0.1] | [0.8,1.0] | [-0.2,0.1] | [0.8,1.0] | [0.0,0.2] | | [0.6,0.8] |
| Netherlands | 1.5 | 1.7 | 0.1 | 0.9 | 0.0 | 2.2 | 0.0 | | 0.6 |
|  | [1.4,1.6] | [1.6,1.8] | [0.0,0.2] | [0.8,1.0] | [-0.1,0.1] | [2.1,2.3] | [-0.1,0.1] | | [0.5,0.7] |
| Belgium | 1.6 | 2.1 | 0.1 | 1.3 | 0.0 | 1.1 | -0.2 | | 0.4 |
|  | [1.5,1.7] | [2.0,2.2] | [0.0,0.2] | [1.2,1.4] | [-0.1,0.1] | [1.0,1.2] | [-0.3,-0.1] | | [0.3,0.5] |
| Austria | 2.1 | 2.1 | 0.2 | 0.9 | 0.0 | 2.0 | 0.9 | | 0.8 |
|  | [2.0,2.2] | [2.0,2.2] | [0.1,0.3] | [0.8,1.0] | [-0.2,0.2] | [1.9,2.1] | [0.8,1.0] | | [0.7,0.9] |
| Switzerland | 0.9 | 1.5 | 0.1 | 0.7 | 0.0 | 1.0 | 0.2 | | 0.0 |
|  | [0.8,1.0] | [1.4,1.6] | [0.0,0.2] | [0.6,0.8] | [-0.2,0.1] | [0.9,1.1] | [0.1,0.3] | | [-0.1,0.1] |
| France | 0.9 | 1.6 | 0.2 | 0.3 | 0.0 | 2.2 | -0.2 | | 0.4 |
|  | [0.8,1.0] | [1.5,1.7] | [0.1,0.3] | [0.2,0.4] | [-0.1,0.1] | [2.1,2.4] | [-0.3,-0.1] | | [0.3,0.5] |
| Spain | 0.6 | 1.1 | 0.1 | 0.0 | 0.0 | 1.5 | 0.3 | | 0.4 |
|  | [0.5,0.7] | [1.0,1.2] | [0.0,0.2] | [-0.1,0.1] | [-0.1,0.1] | [1.4,1.6] | [0.2,0.4] | | [0.3,0.5] |
| Hungary | 1.1 | 2.8 | 0.0 | 1.3 | 0.0 | 1.3 | 0.1 | | 0.9 |
|  | [1.0,1.2] | [2.7,2.9] | [-0.1,0.1] | [1.2,1.4] | [-0.1,0.1] | [1.2,1.4] | [0.0,0.2] | | [0.8,1.0] |
| Poland | -0.1 | 2.5 | 0.0 | 0.6 | 0.0 | 0.5 | 0.5 | | 0.8 |
|  | [-0.2,-0.0] | [2.4,2.6] | [-0.1,0.1] | [0.5,0.7] | [-0.1,0.1] | [0.4,0.6] | [0.4,0.6] | | [0.7,0.9] |
| Lithuania | 0.2 | 2.2 | 0.1 | 0.8 | 0.0 | 1.7 | -0.5 | | 1.4 |
|  | [0.1,0.3] | [2.1,2.3] | [0.0,0.2] | [0.7,0.9] | [-0.1,0.2] | [1.6,1.8] | [-0.6,-0.4] | | [1.3,1.5] |
| Estonia | -0.3 | 2.1 | 0.1 | 1.9 | 0.0 | 2.1 | 0.3 | | 1.1 |
|  | [-0.4,-0.2] | [2.0,2.2] | [0.0,0.2] | [1.7,2.0] | [-0.1,0.2] | [2.0,2.3] | [0.2,0.4] | | [1.0,1.2] |
| Average | 1.0 | 1.7 | 0.1 | 0.6 | 0.0 | 1.4 | 0.1 | | 0.6 |
|  | [0.8,1.1] | [1.6,1.8] | [0.0,0.2] | [0.5,0.8] | [-0.1,0.4] | [1.3,1.5] | [0.0,0.2] | | [0.5,0.7] |

**Appendix table H1. Sensitivity Analyses for change in Relative Risk of mortality and disability on the absolute change in educational inequalities**

1. **Men**

| **Risk Factor** | **Country** | **-40%** | **-20%** | **Main** | **+20%** | **+40%** |
| --- | --- | --- | --- | --- | --- | --- |
| Manual Occ. Father | Finland | -0,5 | -0,6 | -0,7 | -0,8 | -0,9 |
| Lowest Inc. Quintile | Finland | -1,1 | -1,4 | -1,6 | -1,9 | -2,1 |
| Social Contact | Finland | -0,2 | -0,2 | -0,2 | -0,2 | -0,3 |
| Smoking | Finland | -1,1 | -1,2 | -1,4 | -1,5 | -1,6 |
| Alcohol | Finland | 0,2 | 0,2 | 0,1 | 0,3 | 0,3 |
| BMI | Finland | -0,7 | -0,8 | -0,9 | -1,0 | -1,1 |
| Physical Activity | Finland | -0,3 | -0,4 | -0,4 | -0,5 | -0,5 |
| Fruit & Vegetable | Finland | -0,3 | -0,4 | -0,4 | -0,5 | -0,6 |
| Manual Occ. Father | Sweden | -0,5 | -0,6 | -0,7 | -0,8 | -0,9 |
| Lowest Inc. Quintile | Sweden | -0,5 | -0,6 | -0,7 | -0,8 | -0,9 |
| Social Contact | Sweden | 0,0 | 0,0 | 0,0 | -0,1 | -0,1 |
| Smoking | Sweden | -0,9 | -1,0 | -1,1 | -1,2 | -1,3 |
| Alcohol | Sweden | -0,1 | -0,1 | 0,0 | -0,1 | -0,1 |
| BMI | Sweden | -0,9 | -1,1 | -1,3 | -1,4 | -1,5 |
| Physical Activity | Sweden | -0,2 | -0,2 | -0,3 | -0,3 | -0,3 |
| Fruit & Vegetable | Sweden | -0,4 | -0,5 | -0,6 | -0,7 | -0,7 |
| Manual Occ. Father | Norway | -0,5 | -0,7 | -0,8 | -0,9 | -1,0 |
| Lowest Inc. Quintile | Norway | -0,6 | -0,7 | -0,9 | -1,0 | -1,1 |
| Social Contact | Norway | 0,0 | 0,0 | 0,0 | 0,0 | 0,0 |
| Smoking | Norway | -1,2 | -1,4 | -1,5 | -1,6 | -1,7 |
| Alcohol | Norway | 0,2 | 0,2 | 0,1 | 0,2 | 0,3 |
| BMI | Norway | -0,3 | -0,4 | -0,5 | -0,5 | -0,6 |
| Physical Activity | Norway | -0,1 | -0,1 | -0,2 | -0,2 | -0,2 |
| Fruit & Vegetable | Norway | -0,5 | -0,6 | -0,7 | -0,7 | -0,8 |
| Manual Occ. Father | Denmark | -0,8 | -0,9 | -1,1 | -1,3 | -1,4 |
| Low Income | Denmark | -0,7 | -0,8 | -1,0 | -1,1 | -1,3 |
| Social Contact | Denmark | 0,0 | 0,0 | 0,0 | 0,0 | 0,0 |
| Smoking | Denmark | -1,0 | -1,1 | -1,2 | -1,3 | -1,4 |
| Alcohol | Denmark | -0,2 | -0,3 | -0,1 | -0,4 | -0,4 |
| BMI | Denmark | -1,1 | -1,3 | -1,5 | -1,7 | -1,8 |
| Physical Activity | Denmark | 0,3 | 0,3 | 0,4 | 0,4 | 0,4 |
| Fruit & Vegetable | Denmark | -0,7 | -0,9 | -1,0 | -1,1 | -1,2 |
| Manual Occ. Father | UK | -0,6 | -0,8 | -0,9 | -1,0 | -1,1 |
| Low Income | UK | -0,8 | -1,0 | -1,2 | -1,3 | -1,5 |
| Social Contact | UK | 0,0 | 0,0 | 0,0 | 0,0 | 0,0 |
| Smoking | UK | -0,7 | -0,8 | -0,8 | -1,0 | -1,1 |
| Alcohol | UK | 0,0 | 0,0 | 0,0 | -0,1 | -0,1 |
| BMI | UK | -0,7 | -0,8 | -1,0 | -1,1 | -1,2 |
| Physical Activity | UK | -0,3 | -0,4 | -0,5 | -0,5 | -0,6 |
| Fruit & Vegetable | UK | -0,5 | -0,6 | -0,7 | -0,8 | -0,9 |
| Manual Occ. Father | Netherlands | -0,6 | -0,7 | -0,8 | -1,0 | -1,1 |
| Low Income | Netherlands | -0,8 | -1,0 | -1,2 | -1,4 | -1,5 |
| Social Contact | Netherlands | 0,0 | 0,0 | 0,0 | 0,0 | 0,0 |
| Smoking | Netherlands | -0,8 | -0,9 | -1,0 | -1,2 | -1,3 |
| Alcohol | Netherlands | 0,0 | 0,0 | 0,0 | 0,0 | 0,0 |
| BMI | Netherlands | -0,8 | -0,9 | -1,1 | -1,2 | -1,4 |
| Physical Activity | Netherlands | 0,1 | 0,2 | 0,2 | 0,2 | 0,2 |
| Fruit & Vegetable | Netherlands | -0,2 | -0,2 | -0,2 | -0,3 | -0,3 |
| Manual Occ. Father | Belgium | -0,9 | -1,1 | -1,3 | -1,5 | -1,7 |
| Low Income | Belgium | -1,0 | -1,3 | -1,5 | -1,7 | -1,9 |
| Social Contact | Belgium | 0,0 | 0,0 | 0,0 | 0,0 | 0,0 |
| Smoking | Belgium | -1,1 | -1,2 | -1,3 | -1,5 | -1,6 |
| Alcohol | Belgium | 0,2 | 0,2 | 0,1 | 0,3 | 0,4 |
| BMI | Belgium | -0,4 | -0,5 | -0,5 | -0,6 | -0,7 |
| Physical Activity | Belgium | 0,2 | 0,3 | 0,3 | 0,3 | 0,4 |
| Fruit & Vegetable | Belgium | -0,4 | -0,4 | -0,5 | -0,6 | -0,7 |
| Manual Occ. Father | Austria | -1,0 | -1,3 | -1,6 | -1,8 | -2,1 |
| Low Income | Austria | -1,2 | -1,5 | -1,8 | -2,1 | -2,4 |
| Social Contact | Austria | -0,1 | -0,1 | -0,1 | -0,1 | -0,1 |
| Smoking | Austria | -0,7 | -0,8 | -1,0 | -1,1 | -1,2 |
| Alcohol | Austria | -0,1 | -0,2 | 0,0 | -0,2 | -0,3 |
| BMI | Austria | -2,0 | -2,4 | -2,8 | -3,2 | -3,5 |
| Physical Activity | Austria | -1,1 | -1,3 | -1,5 | -1,7 | -1,9 |
| Fruit & Vegetable | Austria | 0,1 | 0,1 | 0,1 | 0,1 | 0,1 |
| Manual Occ. Father | Switzerland | -0,5 | -0,7 | -0,8 | -0,9 | -1,1 |
| Low Income | Switzerland | -1,1 | -1,4 | -1,7 | -1,9 | -2,1 |
| Social Contact | Switzerland | -0,1 | -0,1 | -0,1 | -0,2 | -0,2 |
| Smoking | Switzerland | -0,8 | -0,9 | -1,1 | -1,2 | -1,3 |
| Alcohol | Switzerland | 0,2 | 0,2 | 0,1 | 0,3 | 0,3 |
| BMI | Switzerland | -0,8 | -1,0 | -1,1 | -1,2 | -1,4 |
| Physical Activity | Switzerland | 0,3 | 0,4 | 0,4 | 0,5 | 0,5 |
| Fruit & Vegetable | Switzerland | -0,2 | -0,3 | -0,3 | -0,4 | -0,4 |
| Manual Occ. Father | France | -0,8 | -1,0 | -1,1 | -1,3 | -1,5 |
| Low Income | France | -0,8 | -1,0 | -1,2 | -1,4 | -1,5 |
| Social Contact | France | -0,1 | -0,1 | -0,1 | -0,1 | -0,1 |
| Smoking | France | -0,8 | -0,9 | -1,0 | -1,0 | -1,1 |
| Alcohol | France | -0,2 | -0,2 | -0,1 | -0,3 | -0,4 |
| BMI | France | -0,8 | -1,0 | -1,1 | -1,3 | -1,4 |
| Physical Activity | France | 0,0 | 0,0 | 0,0 | 0,0 | 0,0 |
| Fruit & Vegetable | France | -0,2 | -0,3 | -0,3 | -0,3 | -0,4 |
| Manual Occ. Father | Spain | -0,5 | -0,6 | -0,8 | -0,9 | -1,0 |
| Low Income | Spain | -0,7 | -0,9 | -1,0 | -1,2 | -1,3 |
| Social Contact | Spain | 0,0 | 0,0 | 0,0 | 0,0 | 0,0 |
| Smoking | Spain | -0,4 | -0,4 | -0,5 | -0,5 | -0,6 |
| Alcohol | Spain | -0,1 | -0,2 | -0,1 | -0,2 | -0,3 |
| BMI | Spain | -0,7 | -0,8 | -0,9 | -1,0 | -1,1 |
| Physical Activity | Spain | 0,1 | 0,1 | 0,1 | 0,1 | 0,1 |
| Fruit & Vegetable | Spain | -0,2 | -0,2 | -0,3 | -0,3 | -0,3 |
| Manual Occ. Father | Hungary | -0,4 | -0,6 | -0,7 | -0,8 | -0,9 |
| Low Income | Hungary | -1,6 | -2,0 | -2,3 | -2,7 | -3,0 |
| Social Contact | Hungary | 0,0 | 0,0 | 0,0 | 0,0 | 0,0 |
| Smoking | Hungary | -1,9 | -2,2 | -2,4 | -2,7 | -2,9 |
| Alcohol | Hungary | 0,0 | -0,1 | 0,0 | -0,1 | -0,1 |
| BMI | Hungary | 0,5 | 0,6 | 0,7 | 0,8 | 0,9 |
| Physical Activity | Hungary | -0,2 | -0,2 | -0,2 | -0,3 | -0,3 |
| Fruit & Vegetable | Hungary | -0,4 | -0,4 | -0,5 | -0,6 | -0,6 |
| Manual Occ. Father | Poland | -0,2 | -0,3 | -0,3 | -0,4 | -0,4 |
| Low Income | Poland | -1,6 | -1,9 | -2,2 | -2,5 | -2,8 |
| Social Contact | Poland | 0,1 | 0,1 | 0,1 | 0,1 | 0,1 |
| Smoking | Poland | -0,8 | -0,8 | -0,9 | -0,9 | -1,0 |
| Alcohol | Poland | -0,1 | -0,1 | -0,1 | -0,2 | -0,2 |
| BMI | Poland | -0,4 | -0,4 | -0,5 | -0,5 | -0,6 |
| Physical Activity | Poland | 0,3 | 0,3 | 0,3 | 0,4 | 0,4 |
| Fruit & Vegetable | Poland | -0,1 | -0,1 | -0,1 | -0,1 | -0,2 |
| Manual Occ. Father | Lithuania | -0,3 | -0,4 | -0,5 | -0,5 | -0,6 |
| Low Income | Lithuania | -1,6 | -1,9 | -2,2 | -2,5 | -2,7 |
| Social Contact | Lithuania | 0,0 | 0,0 | -0,1 | -0,1 | -0,1 |
| Smoking | Lithuania | -1,1 | -1,2 | -1,3 | -1,4 | -1,5 |
| Alcohol | Lithuania | -0,8 | -0,9 | -0,5 | -1,2 | -1,3 |
| BMI | Lithuania | -0,4 | -0,5 | -0,6 | -0,6 | -0,7 |
| Physical Activity | Lithuania | 0,6 | 0,7 | 0,7 | 0,8 | 0,9 |
| Fruit & Vegetable | Lithuania | -0,9 | -1,1 | -1,2 | -1,4 | -1,5 |
| Manual Occ. Father | Estonia | -0,5 | -0,6 | -0,8 | -0,9 | -1,0 |
| Low Income | Estonia | -1,2 | -1,5 | -1,8 | -2,1 | -2,3 |
| Social Contact | Estonia | 0,1 | 0,1 | 0,1 | 0,2 | 0,2 |
| Smoking | Estonia | -1,8 | -2,0 | -2,3 | -2,5 | -2,7 |
| Alcohol | Estonia | 0,1 | 0,1 | 0,0 | 0,2 | 0,2 |
| BMI | Estonia | 0,0 | 0,1 | 0,1 | 0,1 | 0,1 |
| Physical Activity | Estonia | 0,5 | 0,7 | 0,8 | 0,9 | 1,0 |
| Fruit & Vegetable | Estonia | -0,4 | -0,5 | -0,6 | -0,7 | -0,7 |
| Manual Occ. Father | Average | -0,6 | -0,7 | -0,8 | -1,0 | -1,1 |
| Low Income | Average | -0,9 | -1,2 | -1,4 | -1,6 | -1,7 |
| Social Contact | Average | 0,0 | 0,0 | 0,0 | 0,0 | 0,0 |
| Smoking | Average | -0,8 | -0,9 | -1,0 | -1,0 | -1,1 |
| Alcohol | Average | -0,1 | -0,1 | 0,0 | -0,1 | -0,2 |
| BMI | Average | -0,7 | -0,8 | -0,9 | -1,0 | -1,1 |
| Physical Activity | Average | 0,0 | -0,1 | -0,1 | -0,1 | -0,1 |
| Fruit & Vegetable | Average | -0,3 | -0,3 | -0,4 | -0,5 | -0,5 |
| **B. Women** | |  |  |  |  |  |
| Manual Occ. Father | Finland | -0,6 | -0,7 | -0,9 | -1,0 | -1,2 |
| Low Income | Finland | -1,1 | -1,4 | -1,6 | -1,9 | -2,1 |
| Social Contact | Finland | 0,0 | 0,0 | 0,0 | 0,0 | 0,0 |
| Smoking | Finland | -1,1 | -1,2 | -1,4 | -1,6 | -1,7 |
| Alcohol | Finland | 0,0 | 0,0 | 0,0 | 0,0 | 0,0 |
| BMI | Finland | -1,2 | -1,5 | -1,8 | -2,0 | -2,2 |
| Physical Activity | Finland | 0,1 | 0,2 | 0,2 | 0,2 | 0,3 |
| Fruit & Vegetable | Finland | -0,6 | -0,8 | -0,9 | -1,1 | -1,2 |
| Manual Occ. Father | Sweden | -0,8 | -1,0 | -1,2 | -1,3 | -1,5 |
| Low Income | Sweden | -0,7 | -0,9 | -1,1 | -1,2 | -1,4 |
| Social Contact | Sweden | 0,0 | 0,0 | 0,0 | 0,0 | 0,0 |
| Smoking | Sweden | 0,0 | 0,1 | 0,1 | 0,2 | 0,2 |
| Alcohol | Sweden | 0,0 | 0,1 | 0,0 | 0,1 | 0,1 |
| BMI | Sweden | -1,1 | -1,4 | -1,6 | -1,8 | -2,0 |
| Physical Activity | Sweden | -0,1 | -0,1 | -0,2 | -0,2 | -0,2 |
| Fruit & Vegetable | Sweden | -0,6 | -0,7 | -0,9 | -1,0 | -1,1 |
| Manual Occ. Father | Norway | -0,9 | -1,1 | -1,3 | -1,5 | -1,7 |
| Low Income | Norway | -1,1 | -1,4 | -1,6 | -1,9 | -2,1 |
| Social Contact | Norway | -0,1 | -0,2 | -0,2 | -0,2 | -0,3 |
| Smoking | Norway | -1,2 | -1,4 | -1,6 | -1,8 | -2,0 |
| Alcohol | Norway | 0,0 | 0,0 | 0,0 | 0,0 | 0,0 |
| BMI | Norway | -1,1 | -1,4 | -1,6 | -1,8 | -2,0 |
| Physical Activity | Norway | -0,2 | -0,3 | -0,3 | -0,4 | -0,4 |
| Fruit & Vegetable | Norway | -0,4 | -0,5 | -0,6 | -0,7 | -0,8 |
| Manual Occ. Father | Denmark | -0,6 | -0,8 | -0,9 | -1,1 | -1,2 |
| Low Income | Denmark | -0,7 | -0,9 | -1,1 | -1,3 | -1,4 |
| Social Contact | Denmark | -0,1 | -0,1 | -0,1 | -0,1 | -0,2 |
| Smoking | Denmark | -0,9 | -1,0 | -1,1 | -1,2 | -1,3 |
| Alcohol | Denmark | 0,0 | 0,0 | 0,0 | 0,0 | 0,0 |
| BMI | Denmark | -1,1 | -1,3 | -1,5 | -1,7 | -1,9 |
| Physical Activity | Denmark | -0,2 | -0,2 | -0,3 | -0,3 | -0,3 |
| Fruit & Vegetable | Denmark | -0,6 | -0,7 | -0,9 | -1,0 | -1,1 |
| Manual Occ. Father | UK | -1,0 | -1,2 | -1,4 | -1,7 | -1,9 |
| Low Income | UK | -0,9 | -1,2 | -1,4 | -1,6 | -1,8 |
| Social Contact | UK | 0,0 | 0,0 | 0,0 | 0,0 | 0,0 |
| Smoking | UK | -0,7 | -0,8 | -0,9 | -1,0 | -1,1 |
| Alcohol | UK | 0,1 | 0,1 | 0,0 | 0,2 | 0,2 |
| BMI | UK | -0,6 | -0,8 | -0,9 | -1,0 | -1,1 |
| Physical Activity | UK | -0,1 | -0,1 | -0,1 | -0,1 | -0,1 |
| Fruit & Vegetable | UK | -0,5 | -0,6 | -0,7 | -0,8 | -1,0 |
| Manual Occ. Father | Netherlands | -1,0 | -1,3 | -1,5 | -1,7 | -2,0 |
| Low Income | Netherlands | -1,1 | -1,4 | -1,7 | -2,0 | -2,3 |
| Social Contact | Netherlands | -0,1 | -0,1 | -0,1 | -0,1 | -0,1 |
| Smoking | Netherlands | -0,7 | -0,8 | -0,9 | -1,0 | -1,1 |
| Alcohol | Netherlands | 0,0 | 0,0 | 0,0 | 0,0 | 0,0 |
| BMI | Netherlands | -1,5 | -1,9 | -2,2 | -2,5 | -2,8 |
| Physical Activity | Netherlands | 0,0 | 0,0 | 0,0 | 0,0 | 0,0 |
| Fruit & Vegetable | Netherlands | -0,4 | -0,5 | -0,6 | -0,6 | -0,7 |
| Manual Occ. Father | Belgium | -1,1 | -1,4 | -1,6 | -1,9 | -2,1 |
| Low Income | Belgium | -1,4 | -1,8 | -2,1 | -2,4 | -2,7 |
| Social Contact | Belgium | -0,1 | -0,1 | -0,1 | -0,1 | -0,1 |
| Smoking | Belgium | -1,0 | -1,1 | -1,3 | -1,5 | -1,6 |
| Alcohol | Belgium | 0,0 | 0,0 | 0,0 | 0,0 | 0,0 |
| BMI | Belgium | -0,8 | -1,0 | -1,1 | -1,3 | -1,4 |
| Physical Activity | Belgium | 0,1 | 0,2 | 0,2 | 0,2 | 0,2 |
| Fruit & Vegetable | Belgium | -0,3 | -0,3 | -0,4 | -0,4 | -0,5 |
| Manual Occ. Father | Austria | -1,4 | -1,8 | -2,1 | -2,5 | -2,8 |
| Low Income | Austria | -1,4 | -1,7 | -2,1 | -2,4 | -2,7 |
| Social Contact | Austria | -0,1 | -0,1 | -0,2 | -0,2 | -0,2 |
| Smoking | Austria | -0,6 | -0,8 | -0,9 | -1,0 | -1,1 |
| Alcohol | Austria | 0,0 | 0,1 | 0,0 | 0,1 | 0,1 |
| BMI | Austria | -1,4 | -1,7 | -2,0 | -2,3 | -2,6 |
| Physical Activity | Austria | -0,6 | -0,7 | -0,9 | -1,0 | -1,1 |
| Fruit & Vegetable | Austria | -0,5 | -0,7 | -0,8 | -0,9 | -1,1 |
| Manual Occ. Father | Switzerland | -0,6 | -0,8 | -0,9 | -1,1 | -1,2 |
| Low Income | Switzerland | -1,0 | -1,3 | -1,5 | -1,8 | -2,0 |
| Social Contact | Switzerland | -0,1 | -0,1 | -0,1 | -0,1 | -0,2 |
| Smoking | Switzerland | -0,5 | -0,6 | -0,7 | -0,8 | -0,9 |
| Alcohol | Switzerland | 0,1 | 0,1 | 0,0 | 0,1 | 0,1 |
| BMI | Switzerland | -0,7 | -0,9 | -1,0 | -1,2 | -1,3 |
| Physical Activity | Switzerland | -0,1 | -0,2 | -0,2 | -0,2 | -0,3 |
| Fruit & Vegetable | Switzerland | 0,0 | 0,0 | 0,0 | 0,1 | 0,1 |
| Manual Occ. Father | France | -0,6 | -0,8 | -0,9 | -1,0 | -1,2 |
| Low Income | France | -1,1 | -1,4 | -1,6 | -1,9 | -2,1 |
| Social Contact | France | -0,1 | -0,2 | -0,2 | -0,2 | -0,3 |
| Smoking | France | -0,2 | -0,3 | -0,3 | -0,3 | -0,3 |
| Alcohol | France | 0,0 | 0,0 | 0,0 | 0,0 | 0,0 |
| BMI | France | -1,6 | -1,9 | -2,2 | -2,5 | -2,8 |
| Physical Activity | France | 0,1 | 0,2 | 0,2 | 0,2 | 0,3 |
| Fruit & Vegetable | France | -0,2 | -0,3 | -0,4 | -0,4 | -0,5 |
| Manual Occ. Father | Spain | -0,4 | -0,5 | -0,6 | -0,7 | -0,8 |
| Low Income | Spain | -0,7 | -0,9 | -1,1 | -1,3 | -1,5 |
| Social Contact | Spain | -0,1 | -0,1 | -0,1 | -0,1 | -0,1 |
| Smoking | Spain | 0,0 | 0,0 | 0,0 | 0,1 | 0,1 |
| Alcohol | Spain | 0,0 | 0,0 | 0,0 | 0,0 | 0,0 |
| BMI | Spain | -1,1 | -1,3 | -1,5 | -1,7 | -1,9 |
| Physical Activity | Spain | -0,2 | -0,2 | -0,3 | -0,3 | -0,3 |
| Fruit & Vegetable | Spain | -0,2 | -0,3 | -0,4 | -0,4 | -0,5 |
| Manual Occ. Father | Hungary | -0,7 | -0,9 | -1,1 | -1,3 | -1,5 |
| Low Income | Hungary | -1,9 | -2,4 | -2,8 | -3,2 | -3,6 |
| Social Contact | Hungary | 0,0 | 0,0 | 0,0 | 0,0 | 0,0 |
| Smoking | Hungary | -0,9 | -1,1 | -1,3 | -1,4 | -1,6 |
| Alcohol | Hungary | 0,1 | 0,1 | 0,0 | 0,1 | 0,1 |
| BMI | Hungary | -0,9 | -1,1 | -1,3 | -1,5 | -1,6 |
| Physical Activity | Hungary | -0,1 | -0,1 | -0,1 | -0,1 | -0,1 |
| Fruit & Vegetable | Hungary | -0,6 | -0,8 | -0,9 | -1,1 | -1,2 |
| Manual Occ. Father | Poland | 0,0 | 0,0 | 0,1 | 0,1 | 0,1 |
| Low Income | Poland | -1,7 | -2,1 | -2,5 | -2,9 | -3,2 |
| Social Contact | Poland | 0,0 | 0,0 | 0,0 | 0,0 | 0,0 |
| Smoking | Poland | -0,5 | -0,6 | -0,6 | -0,7 | -0,7 |
| Alcohol | Poland | 0,0 | 0,0 | 0,0 | 0,0 | 0,0 |
| BMI | Poland | -0,3 | -0,4 | -0,5 | -0,5 | -0,6 |
| Physical Activity | Poland | -0,4 | -0,5 | -0,5 | -0,6 | -0,7 |
| Fruit & Vegetable | Poland | -0,5 | -0,7 | -0,8 | -0,9 | -1,1 |
| Manual Occ. Father | Lithuania | -0,2 | -0,2 | -0,2 | -0,3 | -0,3 |
| Low Income | Lithuania | -1,5 | -1,8 | -2,2 | -2,5 | -2,8 |
| Social Contact | Lithuania | 0,0 | -0,1 | -0,1 | -0,1 | -0,1 |
| Smoking | Lithuania | -0,6 | -0,7 | -0,8 | -0,9 | -0,9 |
| Alcohol | Lithuania | -0,1 | -0,1 | 0,0 | -0,1 | -0,2 |
| BMI | Lithuania | -1,2 | -1,5 | -1,7 | -2,0 | -2,1 |
| Physical Activity | Lithuania | 0,3 | 0,4 | 0,5 | 0,5 | 0,6 |
| Fruit & Vegetable | Lithuania | -0,9 | -1,2 | -1,4 | -1,6 | -1,8 |
| Manual Occ. Father | Estonia | 0,2 | 0,3 | 0,3 | 0,4 | 0,4 |
| Low Income | Estonia | -1,4 | -1,7 | -2,1 | -2,4 | -2,7 |
| Social Contact | Estonia | -0,1 | -0,1 | -0,1 | -0,1 | -0,1 |
| Smoking | Estonia | -1,4 | -1,6 | -1,9 | -2,1 | -2,3 |
| Alcohol | Estonia | -0,1 | -0,1 | 0,0 | -0,1 | -0,2 |
| BMI | Estonia | -1,5 | -1,9 | -2,1 | -2,4 | -2,7 |
| Physical Activity | Estonia | -0,2 | -0,2 | -0,3 | -0,3 | -0,4 |
| Fruit & Vegetable | Estonia | -0,7 | -0,9 | -1,1 | -1,2 | -1,4 |
| Manual Occ. Father | Average | -0,6 | -0,8 | -1,0 | -1,1 | -1,2 |
| Low Income | Average | -1,1 | -1,4 | -1,7 | -1,9 | -2,2 |
| Social Contact | Average | 0,0 | -0,1 | -0,1 | -0,1 | -0,1 |
| Smoking | Average | -0,5 | -0,5 | -0,6 | -0,7 | -0,8 |
| Alcohol | Average | 0,0 | 0,0 | 0,0 | 0,0 | 0,1 |
| BMI | Average | -1,0 | -1,2 | -1,4 | -1,6 | -1,8 |
| Physical Activity | Average | -0,1 | -0,1 | -0,1 | -0,2 | -0,2 |
| Fruit & Vegetable | Average | -0,4 | -0,5 | -0,6 | -0,7 | -0,8 |
